# Liposomal-Based Formulations: A Path from Basic Research to Temozolomide Delivery Inside Glioblastoma Tissue

**DOI:** 10.3390/pharmaceutics14020308

**Published:** 2022-01-27

**Authors:** Roxana-Maria Amarandi, Alina Ibanescu, Eugen Carasevici, Luminita Marin, Brindusa Dragoi

**Affiliations:** 1 TRANSCEND Research Center, Regional Institute of Oncology Iasi, Str. General Henri Mathias Berthelot 2-4, 700483 Iasi, Romania; rpomohaci@iroiasi.ro (R.-M.A.); alinaibanescu@gmail.com (A.I.); eugen.carasevici@gmail.com (E.C.); 2 “Petru Poni” Institute of Macromolecular Chemistry of Romanian Academy, Aleea Grigore Ghica Voda, nr. 41A, 700487 Iasi, Romania; lmarin@icmpp.ro

**Keywords:** glioblastoma, temozolomide, liposome, chitosan, synthesis, characterization

## Abstract

Glioblastoma (GBM) is a lethal brain cancer with a very difficult therapeutic approach and ultimately frustrating results. Currently, therapeutic success is mainly limited by the high degree of genetic and phenotypic heterogeneity, the blood brain barrier (BBB), as well as increased drug resistance. Temozolomide (TMZ), a monofunctional alkylating agent, is the first line chemotherapeutic drug for GBM treatment. Yet, the therapeutic efficacy of TMZ suffers from its inability to cross the BBB and very short half-life (~2 h), which requires high doses of this drug for a proper therapeutic effect. Encapsulation in a (nano)carrier is a promising strategy to effectively improve the therapeutic effect of TMZ against GBM. Although research on liposomes as carriers for therapeutic agents is still at an early stage, their integration in GBM treatment has a great potential to advance understanding and treating this disease. In this review, we provide a critical discussion on the *preparation methods* and *physico-chemical properties* of liposomes, with a particular emphasis on TMZ-liposomal formulations targeting GBM developed within the last decade. Furthermore, an overview on liposome-based formulations applied to *translational oncology* and *clinical trials* formulations in GBM treatment is provided. We emphasize that despite many years of intense research, more careful investigations are still needed to solve the main issues related to the manufacture of reproducible liposomal TMZ formulations for guaranteed translation to the market.

## 1. Introduction

Over the past decade, considerable efforts have been devoted to optimizing treatment for central nervous system (CNS) tumors, particularly glioblastoma multiforme (GBM). In 2016, 330,000 new patients were diagnosed, while the death toll from CNS tumors reached 227,000 [1]. GBM is the most aggressive form of brain cancer, is incurable and has an average survival rate of 12 months [2]. The main challenges raised by this disease are its high degree of genetic and phenotypic heterogeneity, the blood brain barrier, its increased resistance to therapies, and the existence of cancer stem cells [3,4]. In addition, GBM treatment is expensive [5]. For instance, in the United States, the average direct cost per patient for GBM treatment has been estimated at 8500 $/month, mainly consisting of surgery, imaging, and radiotherapy costs. Direct costs including stays and treatments carried out at hospital strongly depend on country, varying from an average of 27,000 $/patient in Sweden to 95,000 $/patient in the US. Indirect GBM costs (work leave, income losses) are usually much higher than direct ones, with an estimated 101,000 $/patient in Sweden and 112,000 $/patient in Spain. The GBM treatment market was estimated at 465 million $ in 2016 and is expected to reach 1 billion $ by 2025, being equally distributed between the US, Europe, Asia, and the rest of the world. Fortunately, drugs which can potentially be used for the treatment of GBM benefit from the orphan status, a fact which encourages companies to develop novel GBM drugs due to various benefits from national and international regulatory agencies, since GBM is considered a rare disease [5]. The current standard treatment of GBM includes surgery, radiotherapy, and chemotherapy. The first line chemotherapeutic drug for GBM is temozolomide (TMZ), a monofunctional alkylating agent. It was first synthesized in 1984 by Stevens [6] and introduced in a clinical trial in 2005 when, combined with radiotherapy, led to a 7.6-month overall survival benefit [7]. TMZ itself is therapeutically inactive. Under physiological conditions, it is converted into its active form 5-(3-methyltriazeno)imidazole-4 carboxamide (MTIC) through hydrolysis. MTIC kills the tumor cells by preferentially methylating DNA at the N^7^ of guanine in guanine-rich regions, but also at O^6^ guanine and N^3^ adenine [8,9,10]. However, the therapeutic efficacy of TMZ under its active form is far from being optimal mainly due to its inability to cross the blood-brain barrier (BBB) and very short half-life (~2 h) which requires a high dose to achieve the desired therapeutic effect. For instance, in the phase I clinical trial, the recommended dose of TMZ was 750–1000 mg/m^2^ given orally, 5 days per week for 4 weeks [11]. It is well documented that a high dose not only causes drug resistance, but also generates side effects, most commonly myelosuppression, neutropenia, and thrombocytopenia. Moreover, it was reported that prolonged TMZ therapy can unevenly facilitate various disorders, including pneumonia, vascular, nervous, or gastrointestinal disturbance [12]. A promising strategy to address all the raised shortcomings is the protection of TMZ by encapsulation in a (nano)carrier. Indeed, the development of nanometer-sized particles for oncological applications has been widely researched in the last years, showing high potential for GBM diagnosis and treatment. Hence, extensive literature exists on the topic of brain tumor-targeting drug delivery systems, the interested reader being directed to several insightful reviews [1,13,14,15]. All these works highlight the fact that the use of nanocarriers has actually overcome the common disadvantages of non-encapsulated drugs, while cell uptake was increased and significant extension of survival rate was achieved. Clearly, strong and well-documented evidence supports the fact that biocompatible nanocarriers, such as liposome- and chitosan-coated liposome-based formulations, are particularly interesting due to their advantages over other transporters. The current review provides a *brief history* of the use of liposome- and chitosan-coated liposome-based formulations in medicine, followed by a focus on their *preparation methods* and *physico-chemical properties*, with a particular emphasis on TMZ-liposomal formulations for GBM developed within the last ~10 years. Features that facilitate transport across the BBB and accumulation in tumors cells will be particularly highlighted. To keep a realistic note on the use of artificial transporters for entering biological systems, the *pitfalls* of these nanocarriers will be equally discussed. Then, an overview on the liposome- and chitosan-coated liposome-based formulations applied to *translational oncology* will be highlighted. The last section of the review will be devoted to past and ongoing *clinical trials* using liposome- and chitosan-coated liposome-based formulations to target GBM. Although there are numerous discussions in the literature on liposome- and chitosan-coated liposome-based formulations in general, encapsulation of TMZ within these transporters is scarcely reported. It appears that this area offers many opportunities for active research. Therefore, we shall provide both a larger context for the selected carriers, and contributions on TMZ-based formulations whenever literature reports are available.

## 2. Brief History and Overview 

**Figure 1:**
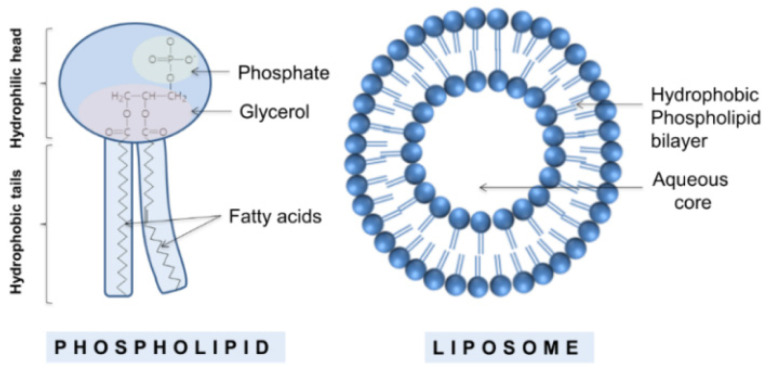
Schematic representation of a liposome.

Liposomes are cell-like membranous structures consisting of phospholipids. Phospholipids are molecules with a polar head and two hydrophobic tails, which spontaneously assemble into vesicles with an aqueous core when exposed to an aqueous environment (Figure 1) [16,17,18].

**Figure 2:**
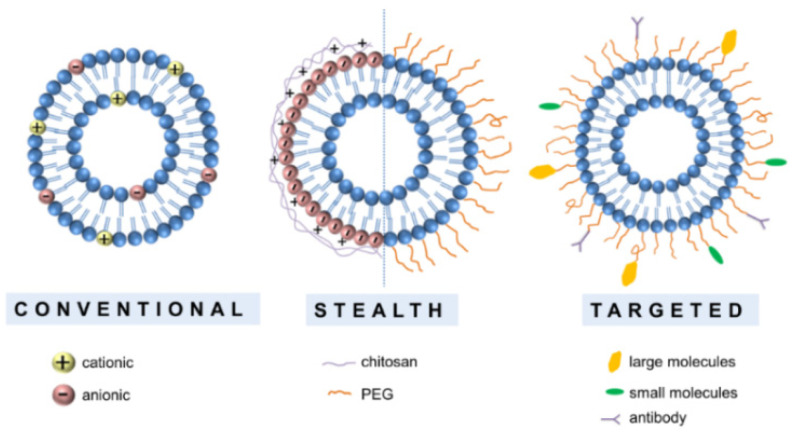
Main types of liposomes.

This particular organization allows for a selective distribution of lipophilic and hydrophilic molecules in the corresponding regions. They were discovered in the 1960s by Bangham at the University of Cambridge, who noticed that lecithin dispersions contain spherulites composed of concentric lamellae with a lipid layer of 4.4 nm [19]. Shortly after, they became interesting “materials” to be used as drug carriers for medical applications, which strongly promoted intense research on their formulation, composition, and properties. For instance, even though they were being prepared from natural lipids at the beginning of their history, a large variety of liposomes made of natural and synthetic anionic and cationic lipids are currently available. In addition, surfactants have been added into their composition, aiming to improve their properties, such as prolonged blood circulation time, high entrapment efficiency, stability, and permeability [20,21]. The large variety of available liposomes allows for their classification into two main classes: traditional and engineered (stealth and targeted) liposomes (Figure 2).

### 2.1. Classical Liposomes

Traditional liposomes made of phospholipids without any additional modifications are usually known as classical or conventional liposomes. According to the overall charge of the phospholipid composition, classical liposomes can be divided into: *(i)* neutral liposomes, *(ii)* cationic liposomes, and *(iii)* anionic liposomes.

Due to their low toxicity, high biocompatibility and biodegradation, conventional liposomes have been extensively used as nanocarriers for a plethora of market-approved drugs, as well as several drugs currently under clinical investigation [22]. For instance, cationic liposomes have also been extensively used for nucleic acid delivery, as they bind to the negatively charged nucleic acids through electrostatic interactions to form lipid complexes (lipoplexes), which prevent nucleic acid enzymatic degradation [23]. Cationic liposomes are particularly advantageous for GBM-aiming formulations due to their ability to engage in favorable electrostatic interactions with the negatively charged BBB and can trigger adsorptive-mediated endocytosis for an increased uptake rate through the BBB [24,25]. However, the major drawback of using cationic liposomes as nanocarriers for brain delivery is their nonspecific uptake to other tissues, as well as their ability to become extensively coated with plasma proteins, which makes them visible to the immune system [26] and accelerates their clearance [27]. For this reason, liposome pre-coating with plasma proteins has been suggested as an effective strategy to enhance liposomal circulation time in vivo [27].

To the best of our knowledge, one of the first studies reporting the encapsulation of TMZ in liposomes was published in 2009 [28]. While investigating the conditions for improving encapsulation efficiency (EE%), the authors noticed an EE improvement following the increase in ammonium sulfate solution concentration and temperature, but an EE decrease when increasing drug-to-lipid ratio. Another study on the encapsulation of TMZ in liposomes was reported in 2010, but without using the term “liposome” for the obtained lipid vesicles [29]. The authors compared the delivery modes of TMZ lipid suspension (inhalation) and free TMZ (intravenous) in a B16F10 melanoma metastatic lung model. They noticed a higher in vitro cytostatic activity for the lipid suspension formulations at low concentrations (i.e., 1 and 10 μM). The results proved that TMZ lipid suspension can be efficiently inhaled for lung cancer therapy.

The next study on the topic of TMZ liposomal encapsulation was published after a gap of several years. Hence, in 2015, Gao et al. developed a liposomal drug delivery system for TMZ which prolonged the half-life, improved the brain targeting ability, and reduced the systemic effects of the drug [9]. The accumulation of TMZ-liposomes in brain was explained by the presence of sorbitol in the liposome composition, which prevented the rejection of liposomes by RES (reticuloendothelial system). The liposomes can get to the brain tissue through the BBB by pinocytosis of endothelial cells. As a result of these processes, the drug concentration in the brain could increase significantly. However, this hypothesis is not supported by appropriate experimental data. Another benefit of the encapsulation of TMZ in liposomes was the reduction of side effects in non-targeted organs, such as heart and lung, resulting from a decreased drug accumulation in those organs, as reflected by the detected TMZ peak concentration (C_max_). All the above-mentioned achievements are particularly important, since they pave the way for developing new anticancer drug formulations.

A debatable study on employing classical liposomes for the encapsulation of TMZ conjugated to gold nanoparticles (NPs) as therapeutic agents against lung cancer via intra-tracheal inhalation was recently published [30]. The main doubt stemming from the reported findings is related to the loading degree of TMZ in phosphate buffer (pH = 7.4). The authors claim using TMZ at a concentration of 6 mg/mL of PBS, while the producer, Cayman Chemical, stipulates that “the solubility of TMZ in phosphate buffered saline (PBS), pH 7.2, is approximately 0.33 mg/mL” with a storage availability of no more than one day [31] and the European Medicine Agency (EMA) provides a solubility of TMZ in sterilized water of 2.5 mg/mL [12]. In addition, it is not clear how much drug was encapsulated in the liposome used for the in vivo investigation and how much TMZ was actually administered through this formulation as compared with the free drug. 

Classical liposomes are well tolerated by the human body, exhibit high biocompatibility, biodegradability, safety and high loading capacity. Their proven effectiveness in experimental models promoted transition toward clinical trials and even marketing approval for many liposomal formulations, which empowered modern therapy by opening up new therapeutic opportunities (vide infra—Section 5 and Section 6) [32,33]. Despite these achievements, traditional liposomes present a number of limitations. For instance, orally administered liposomes are very sensitive to gastrointestinal conditions, i.e., gastric acid, bile salts, and lipases [34], while the intravenous delivery of liposomes is associated with serum protein adsorption, which results in liposome removal by RES, mainly in the liver [35]. When brain is the target of liposomal drug delivery systems, the situation becomes more challenging due to the BBB, formed by the brain microvascular system [36], which separates the bloodstream from the CNS, as well as the P-glycoprotein, the most important efflux transporter associated to the failure of various therapies applied to treat CNS diseases [37]. In addition, nanosized liposomes (<100 nm) tend to attract each other to reduce their surface tension, which causes loss of payload or unwanted mixing [38,39]. Taken together, all these observations severely compromise the application of classical liposomal formulations for a large range of diseases. To overcome these drawbacks and improve the local delivery performance of liposomes, a great deal of effort has been devoted to engineering and optimizing liposomal composition.

### 2.2. Engineered Liposomes

#### 2.2.1. Stealth Liposomes

**Figure 3:**
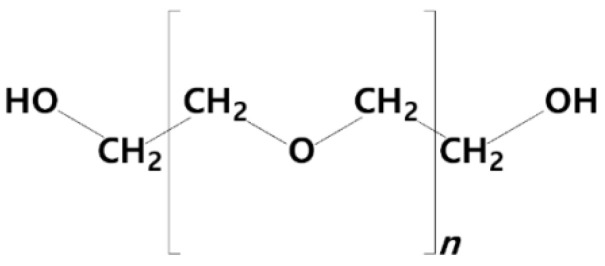
Chemical formula of PEG.

These liposomes are chemically modified by coating conventional liposomes with polymers and polysaccharides, which confers steric stabilization [40]. Of note, cationic macromolecules are preferred for liposomal surface modification, since the overall positive charge also defines the interactions of the engineered (nano)liposomes with biological media components and thus, their fate upon administration and degree of cell internalization. The most common stealth liposomes are those obtained by PEGylation, which was the first design strategy applied to improve liposome performance. It consists of coating the liposome with a layer of polyethylene glycol (PEG, Figure 3), particularly those with a molecular weight (MW) of 1900–5000 Da [41].

It has been shown that the lifetime of such composites in the bloodstream is prolonged, while the fusion of liposomes is prevented [42,43]. In addition, Gref et al. proved even since the 1990s that the circulation time of PEGylated NPs depends on the MW of PEG: the larger the MW, the longer the lifetime of the final nanoparticle [44]. Another study conducted by Chow et al. on PEGylated liposomes led to similar findings, showing that a higher concentration of PEG (6 mol%) improved the pharmacokinetic properties of the final liposomes when compared with a lower concentration of PEG (0.9 mol%) [45]. The enhanced accumulation of these long-circulating carrier systems in tumor cells was associated with the increased leakiness of blood vessels and the reduced lymphatic drainage occurring in tumors, referred to as the enhanced permeability and retention (EPR) effect [46].

The encapsulation of TMZ in stealth liposomes is rather rarely reported. The worked performed by Hu et al. on the co-encapsulation of quercetin and TMZ (1:1 *w*/*w*) in a PEGylated liposome revealed slow drug clearance and prolonged circulation time in vivo upon intragastric administration [47]. Moreover, the prepared formulations exhibited enhanced antitumor effects by killing both drug-sensitive and drug-resistant glioma cells. However, the repeated administration of the quercetin-TMZ liposomal formulations to rats led to a slightly accelerated blood clearance, a phenomenon that perturbed the pharmacokinetic profile of liposomes, which were rapidly eliminated from circulation [48,49]. Still, such a phenomenon is not always observed, which renders the development of TMZ-based liposomal formulations challenging. It is thus recommended that the distribution of the liposomal formulations in the body and the pharmacokinetics of the drugs are investigated when repeated administration is required. In addition, the authors showed that PEGylated liposomes containing both quercetin and TMZ accumulated to a higher degree into the brain when compared with the liposomal formulation containing quercetin only [47]. From a material science point of view, this is somehow confusing, since this finding would suggest a role of the encapsulated TMZ in the accumulation of the liposomal formulation into the targeted organ. On the other hand, the circulation of a carrier in biological fluids, be it a biocompatible liposome, is often viewed as happening in an ideal fashion by the non-medical/biology scientists, which could cause important gaps in understanding what actually happens when biological tissues meet artificially created particles. In addition, according to a schematic illustration of lipid-based NPs (Figure 1E in reference [47]), the core of the particles is decorated with functional groups (-COOH, -SH, -NH_2_) with an unclear origin. Another source of confusion in this work is the appointed terminology. Although “liposome” is frequently used in the paper, the schematic representation of the prepared liposome, showing a lipid core and an “aqueous bilayer”—term which is uncertain, rather indicates the formation of a solid lipid nanoparticle, which is a liposome-like structure [50], but not a liposome. Due to the large diversity of lipid-based (nano)structures, the accurate use of terms is recommended in order to avoid confusions.

Other studies show that when PEGylated liposomes carrying TMZ are delivered directly to the brain, the therapeutic effect of TMZ is increased, while off-target effects associated with TMZ intravenous administration are minimized. For instance, Lin et al. encapsulated TMZ in PEGylated liposomes (8.7 mg/mL TMZ, dipalmitoylphosphatidylcholine—DPPC, cholesterol), which were delivered to the brain by a technique known as convection-enhanced delivery (CED) [51]. The experimental results revealed that mice receiving the TMZ liposomal formulation (5 μL–0.0435 mg/mouse) by CED had no significant increase in survival time when compared with the control group (28.5 days median survival and 25 days median survival, respectively). However, subsequent administration of 5 μL (0.0435 mg/mouse) of the same liposomal TMZ formulation improved animal survival time to greater than 70 days, while a single 10 μL–0.087 mg/mouse resulted in similar survival time as the single 5 μL dose (35 median survival days). Yet, this study failed to include a comparison with the survival rate of mice being administered TMZ-liposomes in a systemic fashion or with other similar studies, making it difficult to appreciate the obtained results at their real value. On the other hand, no systemic toxicity or brain damage were noticed after a three day-administration of the TMZ liposomal formulation. Within the work, this outcome is compared with the administration of CED-liposomal cisplatin, for which neurotoxic effects were shown to appear 10–14 days after administration. However, the comparison is rather unfair, since no results for an investigation at similar periods of time is provided for liposomal TMZ.

The work carried out by Nordling-David et al. on the delivery of TMZ encapsulated in a PEGylated liposomes (1.7 mg/mL TMZ, 1,2-distearoyl-sn-glycero-3-phosphocholine—DSPC and cholesterol) through CED showed a similar effect [52]. Although the results showed no significant difference in cell viability between liposomal TMZ and free TMZ at the highest examined concentration (2 mM) after 24 and 48 h, animals treated with the TMZ liposomal formulation survived ~22% longer than those treated with free TMZ. Moreover, TMZ-liposomes significantly decreased the edema volumes 7 days after providing treatment to animals. The limitation of this study would be the amount of administered TMZ, since the authors used 0.15 μg/g (1 mg/mL; 3 μL/min over 15 min up to a total volume of 45 μL), which is much lower than the tolerated dose of 50 μg/g administered for 5 consecutive days, as reported elsewhere [53]. This is also consistent with the authors’ claim that a higher dose would be more appropriate for the liposomal delivery system in comparison to a drug solution, in terms of tumor inhibition and toxicity profile.

Vanza et al. encapsulated TMZ in a PEGylated liposome made of soybean phosphatidylcholine (PC), dipalmitoyl phosphatidylcholine (DPPC), dipalmitoyl phosphatidylcholine-sodium (DPPG), egg lecithin, cholesterol, and 1,2-distearoyl-phosphatidylethanolamine-methyl-polyethyleneglycol conjugate-2000 (Na^+^ salt) [2]. The results revealed a prolonged release of TMZ from PEGylated liposomes up to 800 min, as compared with free TMZ, which was released in 90 min, according to in vitro studies. In addition, in vivo pharmacokinetic experiments showed a decrease in the clearance of TMZ-encapsulating PEGylated liposomes (TMZ-PL) by 1.5 fold, as compared with free TMZ. Still, the t_1/2_ value of 0.369 h for TMZ-PL was not considerably higher than that of free TMZ (0.39 h in blood). Interestingly, the lifetime of TMZ-PL in the brain was much higher than that of free TMZ (2.575 h vs. 0.42 h). Identification of TMZ after ~2.6 h, together with the total exposure to TMZ and mean residence time in the brain was attributed to the “stealth properties” of the chemically modified liposomes. The conclusions of this study, however, seem incomplete due to several reasons. On the one hand, the results would have been more accurate if classical liposomes were used for comparison while, on the other hand, strong evidence exists that PEGylated liposomes, *per se*, are not capable of crossing the BBB [54]. Despite its success in certain aspects [2], this work still lacks evidence regarding the amount of PEG used to coat the liposomes, particularly since the coating degree is a critical factor in the bio-distribution of the final liposomal product. In addition, the quaternary formulation of the liposomes raises questions related to in vivo applications, since it has been previously shown that defects created in the lipid bilayer of multicomponent liposomes decrease liposome stability in the presence of plasma proteins and biomembranes [55].

**Figure 4:**
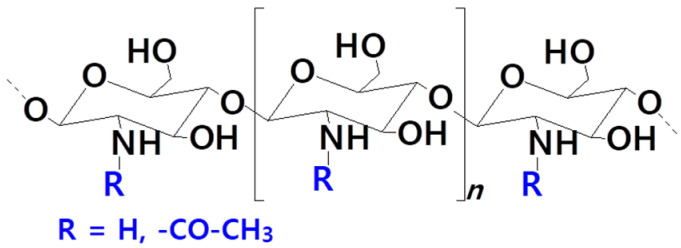
Chemical structure of chitosan.

To fulfill the requirements of biocompatibility, biodegradability, and low toxicity of nanocarriers for medical applications, the employment of polysaccharides for coating the liposomal surface is an excellent strategy. Several research groups have been working on the design of liposomes decorated with *chitosan*, also called *chitosanylated liposomes*, aiming to improve their properties, stability in particular. Apart from the above-mentioned mandatory properties of nanocarriers, the rationale behind using chitosan for liposome formulation resides in its mucoadhesive properties, which increases the penetration of macromolecules through nasal and intestinal barriers [56]. Chitosan, produced by the deacetylation of chitin, is a linear cationic polysaccharide composed of D-glucosamine and N-acetyl-D-glucosamine units linked through a β (1 → 4) linkage (Figure 4) [56,57].

Its degree of deacetylation, which reflects the glucosamine residues, ranges from 30 to 95%, while its average molecular mass is within the range of 126–1000 kDa; both properties are essential for the quality of the final material [57,58,59,60]. For liposomal formulations, the cationic chitosan is adsorbed on the negatively charged surface of liposomes through electrostatic interactions, forming a positive layer around it, a stable chitosan-based network entrapping the liposomes [61,62,63]. This strategy prevents liposome fusion and unintentional payload leakage under both storage and physiological conditions [64]. Albeit the promising properties of chitosan, the number of papers reporting liposomes decorated with chitosan is scarce, most of them being published in the context of controlled drug release and vaccine delivery. The delivery of these carriers covers oral [65], intranasal [66], and transdermal [67] ways, and they are promising candidates for a large range of medical applications including wound healing, vaccine delivery, antimicrobial, and antiviral activity, diabetes, Parkinson’s disease, cancer, etc., [56].

To the best of our knowledge, no previous research has investigated the potential of chitosanylated liposomes as carriers for TMZ delivery, especially for brain cancer. Therefore, this is a research area with a great potential which needs to be addressed in order to develop a new generation of nanocarriers able to improve passage through the most challenging biological barrier, the BBB. In support of this idea, and to highlight the benefits of embedding therapeutic molecules in chitosan-decorated liposomes, a brief overview of the anticancer agents encapsulated in such carriers is provided. Hence, siRNA against breast cancer [68], ursolic acid against cervical cancer [69], butyric acid against hepatocellular carcinoma [70], fluorouracil against colorectal cancer [61], doxorubicin [62], and piperine [71] are among the first antitumor drugs explored as chitosan-based liposomal formulations.

The first study on chitosan-coated liposomes carrying a therapeutic agent for cancer treatment was published by Salva et al. in 2015 [68]. They co-incorporated siHIF1-α and siVEGF, which are siRNAs against HIF1-α and VEGF, into chitosan-coated liposomes (chitosan deacetylation degree of 75–85% and MW of 75 kDa; anionic liposome: PC + cholesterol + dicethylphosphate, cationic liposome: PC + cholesterol + stearylamine) for their co-delivery to breast cancer cells (MCF-7 and MDA-MB-435) under hypoxic conditions aiming to obtain lower cytotoxicity, higher transfection and silencing efficiency. The authors noticed a biological effect of the siRNA dependent on liposomal composition. Hence, the co-delivery of siVEGF and siHIF1-α in a cationic or chitosan-coated anionic liposome increased the suppression level of VEGF expression, as compared with the individual delivery of the two silencing RNAs. Of note, the inhibition effect on both tested cell lines was the highest for the chitosan-based formulation, obviously highlighting the advantage brought by chitosan for the development of highly efficient delivery vehicles for anticancer therapeutic agents.

Ursolic acid, a natural pentacyclic triterpene with anticancer activity, poor water solubility, low bioavailability, and short plasma half-life, was also loaded in anionic and chitosan-coated liposome made of soybean PC and cholesterol [69]. HeLa cells treated with ursolic acid loaded in liposomes (soybean PC + cholesterol) and liposomes decorated with chitosan (deacetylation degree of 92%) exhibited up to 69.51% and 76.46% cell growth inhibition, respectively, while the free drug that inhibited the growth of only 55.14% of cells. Hence, the adsorption of chitosan on the anionic liposomes greatly increased the observed biological effect, accomplishment which was attributed to the pH-responsive drug releasing behavior induced by the presence of chitosan in the drug formulation. This study is the only one among those reporting on the delivery of anticancer agents through chitosan-modified liposomes that provided the in vivo antitumoral activity of the complex formulation. Therefore, the antitumor activity of the ursolic acid formulations was evaluated employing U14 cancer cell xenograft-bearing CD-1 mice. Notably, ursolic acid loaded in chitosan-modified liposomes suppressed tumor growth more efficiently than the free drug or drug encapsulated in unmodified liposomes. The authors associated these results to an enhanced accumulation of ursolic acid at the tumor site and improved release in tumor cells based on the EPR and pH-response due to chitosan. Albeit finding a lower concentration of ursolic acid in the spleen and liver when using chitosan-coated liposomes as compared with free drug or drug encapsulated in uncoated liposomes, quite a high amount had been eliminated by the two immunoregulatory organs. Nevertheless, the remaining chitosan drug formulation was enough to significantly increase the amount of drug at the tumor site by at least four times when compared with the free drug.

The next four papers present the results of cell-based in vitro studies and demonstrate an improved efficiency of the drug encapsulated in the chitosan-modified liposomes, when compared with the free drug.

Quagliariello et al. prepared biodegradable and biocompatible liposomes encapsulating butyric acid, coated or uncoated with chitosan (deacetylation degree not provided) in order to increase both human cancer HepG2 cell uptake and drug internalization [70]. The experimental results clearly revealed that the butyric acid-loaded liposomes coated with chitosan exhibited the best internalization capabilities and considerable higher cytotoxicity than both uncoated liposomes and free drug.

Remarkably, results of in vitro release studies and in vitro cell-based assays revealed an increased efficacy of 5-fluorouracil (5-FU) encapsulated in chitosan-decorated liposomes (chitosan: deacetylation degree of 92%; liposomes: soybean lecithin—>94% PC + cholesterol or soybean lecithin + cholesterol + dicetyl phosphate) in comparison with the drug encapsulated in conventional liposomes [61]. The authors noticed decreased cell viability and an enhanced cytotoxic effect of the 5-FU chitosanylated formulation against HT-29 cells, results which were attributed to the presence of the chitosan layer.

A valuable comparative study of doxorubicin (DOX) included in anionic liposomes (egg yolk lecithin and cardiolipin) and chitosan-modified liposomes (deacetylation degree of 85%) was recently published by Grozdova et al. [62]. In addition, they investigated the role of the structural organization of chitosan, that is, linear and cross-linked with sulfate anions, on cell viability (drug-sensitive MCF-7 and drug-resistant OVCAR-8 cell lines) and the ability of encapsulating liposomes to potentiate the therapeutic efficacy of DOX. First, the authors were interested in evaluating the influence of these two types of chitosan toward the activity-free DOX. They observed that linear chitosan exhibited slight toxicity on MCF-7 cells, while cross-linked chitosan had no toxicity, and both types of chitosan exhibited no toxic effect on the OVCAR-8 cells. Hence, the positively charged cross-linked chitosan was chosen as a cationic scaffold for assembling the negatively charged DOX-loaded liposomes. Interestingly, the toxicity of DOX toward drug-resistant cells was increased when complexed with cross-linked chitosan, obviously contrasting with linear chitosan, results which could be attributed to the chemosensitizing properties of cross-linked chitosan, which are currently under debate [62]. However, this finding is crucial for the design of nanocarriers targeting multidrug resistant cells, as is the case of GBM cells, which achieve this effect through the p-glycoprotein (P-gp), a multidrug resistance protein. As such, anionic liposomes containing DOX were decorated with cross-linked chitosan [62]. For this step, two kinds of complexes were prepared, namely a slightly positive one with the electrophoretic mobility of +0.16 ± 0.1 (μm/S)/(V/cm) and a negatively charged one with the electrophoretic mobility of −0.9 ± 0.2 (μm/S)/(V/cm). Remarkably, the cross-linked chitosan coating on the liposomes carrying DOX, which accessed the cell by endocytosis, delivered a 4–5 times higher amount of DOX to OVCAR-8 cells, when compared with uncoated liposomal or free DOX. Moreover, the released DOX entered the nuclei, a phenomenon typical for free DOX.

The water-insoluble piperine encapsulated in liposomes (PC and cholesterol) stabilized with chitosan (deacetylation degree of >95%) exhibited an increased cytotoxic effect, 1 mM piperine included in chitosan-loaded liposomes killing 80% of MCF cells, as compared with 2 mM free piperine, which killed only 50% of cells [71]. Yet, this study suffers from the lack of relevant conclusions, since the in vitro drug release study showed the lowest release capacity of the free drug, a phenomenon attributed to the drug’s water insolubility, whereas the antioxidant capacity was found to be similar for all tested formulations: free drug, liposome, and chitosan-coated liposome. The results discussed in this study, however, encourage performing in vivo experiments on healthy and tumor-bearing animals to provide data regarding the systemic circulation time of all piperine-based formulations.

The above-presented results undoubtedly show that stealth liposomes, namely PEGylated and chitosanylated liposomes, generally overcome many of the drawbacks of classical liposomes. However, they also have some limitations, particularly PEG-coated liposomes, such as low-cell uptake, poor selectivity for tumor cells and even accelerated blood clearance upon successive injections. For chitosanylated liposomes, blood circulation time is difficult to evaluate due to insufficient evidence from in vivo studies. However, the report of Wang et al. shows that chitosan holds great promise for pushing on the development of liposomes decorated with this cationic polysaccharide [69]. In addition, the discussed studies somewhat reveal a targeting ability of chitosan, being able to differentiate between normal and tumor cells to a certain extent. Moreover, chitosan, as a biocompatible carrier, is a feasible solution for treatment of brain cancer due to sidestepping the limitations imposed by BBB. Indeed, it was recently showed that chitosan has the ability to cross this barrier not only due to its high biocompatibility, but also due to its positive surface charge, which favors the transport through BBB by adsorptive-mediated transcytosis [72,73,74,75]. It can therefore be stated that chitosanylated liposomes are a transitory strategy toward the next level of engineered liposomes, namely targeted liposomes.

#### 2.2.2. Targeting Liposomes

Selectivity, targeting, drug release and uptake at the tumor site are still the key challenges that limit the efficacy of cancer therapy. These properties are mainly aimed at reducing side effects of the free drug, lowering doses, maximizing therapeutic effect and, last but not least, achieving personalized therapy for a single or a group of oncological patients. To fulfill this goal, liposomes or any other (nano)carriers need not only to be “invisible” or “stealth”, but should also be capable of specifically recognizing tumor cells through particular overexpressed receptors or other biologically relevant molecules. The possibility for the surface modification of these carriers has led to the new concept of “targeting”. Actually, this concept is more complex, as it can be divided into two main categories, which are active targeting (AT) and environmentally responsive targeting (ERT) [15]. AT refers to the specific binding to overexpressed receptors via targeting ligands, such as antibodies and peptides. Yet, this is related to improving target cell recognition and target cell uptake, but does not necessarily imply an enhanced overall tumor accumulation [76]. ERT is more complex than AT, since it takes advantage of *(i)* the local tumor microenvironment, such as pH, temperature, reactive oxygen (nitrogen) species, ionic strength, transition metal ions, metabolites, and *(ii)* the exposure to external stimuli, such as light, ultrasounds, heat, and magnetic field. The design of liposome-based nanomedicines in line with ERT holds great promise for the future of cancer therapy, since the selective delivery of the drug at the tumor site is maximized, while the toxic off-target effects are minimized [15,76,77]. In the light of this, mono- and multifunctional-targeting liposomes triggering one, two or several of the local and external stimuli, with optimized therapeutic performance, have been already developed. Nonetheless, this direction is still in its infancy, at a basic research level, because the complexity of the chemical modification of liposomes limits their large-scale manufacturing and quality control [15]. In addition, the synthesis of a multifunctional liposome, so that it is completely specific with regard to stimuli-responsive drug release, is challenging [76]. When discussing this idea in the context of developing targeting liposomes for TMZ delivery for GBM, the specific literature is not yet very generous.

Apart from the challenges mentioned above, the development of multifunctional liposomes carrying TMZ with anti-glioma activity is even more difficult because passage through the BBB has to be considered. A first attempt for the design and synthesis of a more efficient vehicle for TMZ for GBM treatment was reported by Kim et al. in 2015 [78]. The authors synthesized a “dual-targeting” nanomedicine made of a cationic liposome (1,2-dioleoyl-3-trimethylammonium propane—DOTAP and dioleolylphosphatidyl ethanolamine—DOPE) encapsulating TMZ, decorated with anti-transferrin receptor single-chain antibody fragments (TfRscFv), which both favored crossing the BBB and targeted cancer cells. The in vitro experimental results obtained on U87R, a highly TMZ-resistant subclone of the U87 cell line, revealed that the synthesized nanomedicine (scL-TMZ) was capable of killing GBM cells much more effectively than free TMZ. This result indicates a high degree of internalization of the engineered liposome via the cell surface TfR for effective drug delivery. Yet, the control non-glioma cells were also more sensitive to the nanomedicine when compared with the free drug, which indicates a low selectivity. However, the ability to specifically recognize tumor cells, to internalize and to discharge the payload was demonstrated by an in vivo study on mice bearing U87-luc2 cells. The mice were treated twice/week for 5 weeks and the tumor size was monitored by MRI. Animal survival was monitored for 51 days. Tumor shrinkage was noticed on day 19 (the 6th i.v. injection) for both free TMZ and targeting liposomal formulation, but with an obviously enhanced growth inhibitory effect in the latter case. Furthermore, the tumor growth was slower in mice treated with free TMZ, but discontinuing treatment reactivated tumor growth. Remarkably, treatment with the liposomal formulation of TMZ kept the tumor size constant, effect which was preserved for almost three weeks after treatment completion. The authors also investigated the in vivo ability of the TMZ liposomal formulation to overcome drug resistance in mice bearing subcutaneous T98G xenograft tumors. The animals received i.v. injections for five days of either 200 mg/m^2^/day free TMZ or 75 mg/m^2^/day of scL-TMZ. These results confirmed the previous in vitro results, namely that the free drug failed to eradicate the tumor, which continued to grow after treatment was stopped, while the liposomal formulation of TMZ reduced the tumor by 55% after 18 days of treatment. This result reinforces the idea that liposomes decorated with TfRscFv exhibit a dual function: crossing the BBB by targeting the normal endothelial cells of the BBB and reaching the tumor cells. Moreover, a reduced toxicity (hematology, liver, heart) was observed, which indicates a selectivity of the liposomes for the tumor cells, followed by high tumor uptake. Yet, no information on the tumor evolution and animal survival after 18 days of treatment or treatment interruption was provided, which raises uncertainties in view of real patient treatment. This aspect is particularly important, since the fate of nanomedicines entering systemic circulation is shaped by blood components, especially proteins.

To address the problem of protein adsorption while targeting brain cancer cells, Arcella et al. encapsulated TMZ in four cationic liposome (CL) formulations, namely CL1 (DOTAP/cholesterol), CL2 (DOTAP/DOPE), CL3 (DC-Chol/DOPE), and CL4 (DC-Chol/cholesterol) that were loaded with TMZ, followed by incubation with human plasma [55]. In this systematic study, they demonstrated that certain proteins fingerprints (e.g., vitronectrin, apolipoproteins, β3 integrin and vitamin K-dependent protein) in the layer (the so-called “corona”) formed around liposomes upon exposure to biological media can exhibit natural targeting properties against cancer cells. Moreover, it was proved that a higher concentration of these proteins is more favorable for targeting human cervical cancer cell line (HeLa) and human prostate cancer cell line (PC3). Vitronectin, a multifunctional glycoprotein present in blood and the extracellular matrix [79], specifically binds to α_V_β_3_ integrin receptors overexpressed in many solid tumors and tumor vasculature, such as metastatic ductal carcinoma cells [55]. NPs coated with an apolipoprotein-enriched corona proved to be promising candidates for brain drug delivery when compared with other nanoparticle systems. Still, the targeting property is strongly dependent on the liposomal composition as well. The results revealed that the CL2 and CL3 formulations, which were associated with the highest amount of apolipoproteins in the protein corona upon contacting the human plasma, were the most favorable for TMZ delivery to the brain. When TMZ-CL2 was administered to U87 cells, the antitumor efficacy increased by a factor of five compared to corona-free cationic liposomes. The ability to bind to overexpressed receptors on the BBB (e.g., scavenger receptor class B, type I and low-density lipoprotein receptor) was demonstrated by in vitro experiments as well, using the commonly employed human umbilical vein endothelial cells (HUVEC). The authors found that the CL formulation bearing the highest levels of targeting fingerprints exhibited the ability to bind receptors overexpressed at the BBB.

In an attempt to improve the delivery of TMZ at the tumor site and moreover to target the origin of the tumor initiation, that is, stem cells (SCs), Kim et al. prepared a dual-targeting immunoliposome carrying TMZ by using angiopep-2 (An2) and an anti-CD133 monoclonal antibody (CD133 mAb) for BBB transcytosis and specific delivery to GBM stem cells (GSCs), respectively [80]. The in vitro results obtained on U87MG GSCs revealed an increased cytotoxicity of the liposomal formulation of TMZ as compared to free TMZ and even simple TMZ-liposomes. Specifically, the biological activity of the targeted liposome was 425- and 181-fold higher compared with that of free TMZ and non-targeted TMZ-liposomes. The ability to cross the BBB was studied on the BBB kit^TM^ (brain astrocytes, pericytes, and endothelial cells). Chemical modification of the external surface of PEGylated liposomes with An2 proved to be a proficient strategy to cross the BBB, as opposed to modification with CD133 only. Yet, no comparison with free liposomes is provided in this case, which affects the accurateness of the comparison. The in vivo efficacy of the targeted TMZ liposomal formulation was demonstrated on a GBM mouse orthotopic xenograft model by an increased median survival time of 49.2 days, unlike the free TMZ and bare liposome-TMZ for which the survival rates were of 23.3 and 36.5 days, respectively. In addition, the toxicity of both free TMZ and targeted liposomal formulation of TMZ was negligible at doses of 5 and 10 mg/kg in both cases. Considering that toxicity is one of the promoters of TMZ encapsulation in a (nano)carrier, the results could be associated with a low dose of injected TMZ, yet it is not clear whether the delivered amount refers to the entire nanomedicine or to the encapsulated TMZ amount, in the case of liposomal drug. Another aspect that should be considered when such a comparison is made is the metabolic fate of TMZ in a physiological environment. It is well acknowledged that the active form of TMZ is the hydrolyzed product obtained under physiological conditions [8]. Hence, the results could be inaccurate, since a delay in the effect of free and encapsulated TMZ should be considered and perhaps should be reflected by the observed toxicity level. When such metabolic conversions of the drug are not considered during the investigation, it can cause confusion in the research community. Another source of inaccuracy identified in this work is the preparation and physical properties of the liposomes, as will be discussed below in the corresponding sections. Apart from the weaknesses of this work, the attempt to functionalize liposomes or any other biocompatible (nano)carriers with molecules able to recognize specific protein receptors (over)expressed on endothelial cells of the BBB and glioma cells is indeed an approach with real potential in GBM treatment.

Recently, Lam et al. published an article on the treatment of GBM with TMZ and bromodomain inhibitor JQ1 encapsulated in a PEGylated liposome functionalized with transferrin [81]. First, the authors screened two ligands (transferrin—Tf and folate— Fol) with the aim of assessing their ability to cross the healthy BBB in mice. Non-functionalized PEGylated liposomes and hemagglutinin (Hg)-modified PEG-liposomes were employed as negative controls. The experimental results indicated a total uptake of 1.7 and 0.9% of the injected dose for Tf- and Fol-modified carriers in the brain at 24 h upon injection, while the number in negative controls was negligible. To confirm the integrity of the brain microvessels, a leakage test was also performed, which confirmed the absence of any outflow. Moreover, Hg-liposomes injected intravenously failed to be uptaken by the endothelial cells of brain microvessels, as compared with the Tf-liposomes, which were successfully transported across the endothelium. Once the ability to cross an intact BBB was proven, the next step was to investigate the transcytosis ability of two glioma cells, namely, human U87MG, and murine GL261 cell lines against Tf-liposomes. Flow cytometry results showed that after 24 h of incubation, 13% of Cy5.5-labelled Tf-liposomes were distributed in the intracellular compartments, compared to <1% Cy5.5-PEGylated liposomes. It is thus obvious that Tf is critical for the internalization of liposomes in these two cell lines. The ability to cross the BBB and accumulate at the tumor site in vivo was also proved in mice bearing either U87MG or GL261 tumors. Interestingly, the authors noticed the accumulation and uptake of Tf-NPs on the surface of U87MG and GL261 tumors, while PEG-liposomes failed to demonstrate accumulation or uptake in the two tumor models. These results pointed out that the functionalization with transferrin is a requisite to achieve passage through BBB and deliver the drug to glioma cells. Moreover, no difference between the therapeutic effect of free TMZ and TMZ encapsulated in non-targeted PEGylated liposomes (2 mg/kg TMZ) on tumor-bearing mice was noticed despite the stealth properties of the carrier system. Furthermore, the effects observed upon the administration of Tf-liposomes to mice confirmed the in vitro results on cells, for which the role played by Tf in TMZ cell uptake were evident. Nevertheless, this study showed that only a small part of the PEGylated liposomes delivered to the brain could reach the target, unlike other works reporting the successful delivery of most part of the amount of the drug-loaded nanocarrier [2,47,80]. Therefore, despite many years of intense research, the role of PEG in targeting the brain remains controversial, highlighting the need of additional and more careful research when PEG-based (nano)carriers are at the core of drug delivery for treating human diseases. This recommendation should be followed to realistically improve the efficiency of the delivery system while ensuring its safety once inside the body, as well as the cost effectiveness of nanoparticle-based medicines.

Another study focused on the development of targeted liposomes for brain cancer treatment was published by Gabay et al. [82]. The proposed system was composed of PC-based liposomes functionalized with a targeting peptide made of a short sequence of 5 amino acids (RERMS) present in the amyloid precursor protein (APP), which can effectively cross the BBB. First, the BBB permeability of the conjugated liposomes was studied in vitro on a BBB model consisting of primary endothelial cells collected and cultured from porcine brains. After 24 h of exposure, most of the non-targeted liposomes remained in the pre-BBB compartment, while 60% of the targeted liposomes did not pass the barrier. The in vivo results on mice bearing U87 tumors showed a survival period of 35 ± 5 days upon treatment with TMZ incorporated in targeted liposomes (4 mg/kg TMZ), and only 25 ± 1 days for those receiving free TMZ.

The most recent study on TMZ-liposomes targeting the BBB was published by Zhang et al. [83]. The authors developed a glucose-functionalized liposome (gLTP) made of TMZ and pro-apoptotic peptide (PAP) to achieve synergistic efficacy toward GBM. The experimental results showed that the gLTP can easily penetrate the BBB via the glucose–GLUT1 pathway and discharge the payload in the cells. Moreover, due to the presence of PAP, which has the ability to destroy the mitochondria of the tumor cells, the anti-tumor efficacy of TMZ was increased.

**Figure 5:**
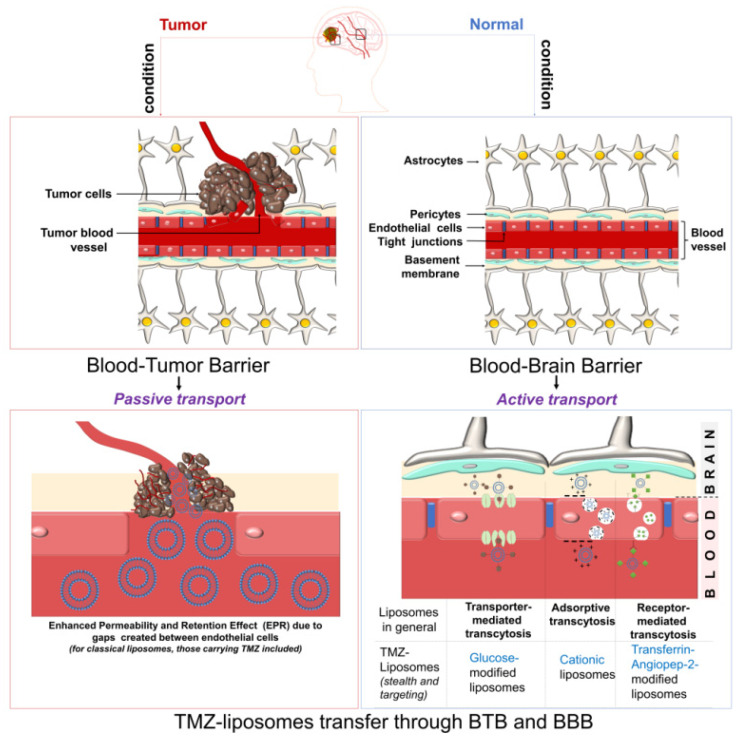
Strategies for TMZ-liposomes crossing the BBB and BTB.

As mentioned above, the main challenges of targeting glioma cells are the biological barriers, especially BBB and blood-tumor barrier (BTB). Yet, the achievements discussed in the papers reviewed in this work prove the successful passage of TMZ-liposomes through these two barriers and discharge of cargo in the tumor cells. On the other hand, the transport of liposomes through these two barriers is not necessarily related to the cargo, but rather to the composition of liposome. Considering the chemistry of liposomes designed for TMZ encapsulation and the strategies through which liposomes for brain tumors diagnosis and therapy pass across these two barriers, several approaches can be considered for the passage of TMZ-liposomes through BBB and BTB (Figure 5) [25,84,85,86].

The first and most used strategy by which liposomes, including those carrying TMZ, pass through the BBB is the one that takes advantage of the vulnerability of the vascular network in the tumor microenvironment. Normally, the endothelial cells of healthy blood vessels are tightly connected, severely limiting the transfer of molecules between the blood and brain. However, under tumor conditions, this structural and functional integrity is lost, and the gaps created in the endothelial wall allows for the access of foreign molecules, particles, and other entities to the brain by the so called EPR effect. This effect is also reinforced by the compromised lymphatic drainage around the tumor. This strategy, also known as *passive targeting*, is mainly advantageous for the classical liposomes without chemical modification of the surface. In addition, tumor angiogenesis generates a second barrier, the BTB, which delimits the tumor tissue from these new vessels that support the growth of the tumor. Unlike BBB, the BTB is more accessible to liposomes by the EPR effect due to the inter-endothelial voids, allowing thus the free passage of liposomes. Then, the liposomes are internalized via highly expressed receptors on tumor cells, such as epidermal growth factor receptors. The second strategy of crossing the BBB is *active targeting*. This type of transport is more effective than passive targeting because it allows for a more selective uptake of liposomesdue to the use of the chemical particularities of the undamaged membrane to be crossed. Depending on the facilities provided by the membrane, the liposomes can be designed to trigger a particular pathway. For instance, in the case of engineered TMZ-liposomes, the following types of active transport can be proposed (Figure 5):

- Transporter-mediated transcytosis, when glucose is used as the transporter [83];
- Adsorptive transcytosis, when the transport is based on the electrostatic interactions between the negatively charged membrane and the positively charged liposomes (cationic liposomes) [47];
- Receptor-mediated transcytosis, strategy fulfilled out by decorating the targeting TMZ-liposome with Tf, An2 [78,80].

Although several ways of transport through the BBB are possible, it can be stated that a very strict delimitation between them is very difficult to make. The high complexity of the biological environment makes the overlap of these strategies possible, especially for engineered liposomes.

The postulation that can be truthfully derived from the studies discussed above is that the specific binding between targets and ligands has a real potential to improve the selectivity of multifunctional (targeted) liposomes to cancer cells by increasing the accumulation and uptake at the tumor sites. However, the complexity of such systems requires a great deal of effort to prepare them in a reproducible manner, which severely impacts their large-scale production and application in clinical practice. Moreover, the use of liposomes to encapsulate a drug, in this case TMZ, is mainly to protect it against degradation, to reduce its side effects on non-targeted organs, and finally to improve the drug efficacy at lower doses. On the other hand, the limitations and pitfalls of the carrier, in this case, liposomes, should be also considered in order to keep a realistic view on the designed nanoformulations for cancer diagnosis and therapy. This topic has been well documented and discussed by Lammers et al. in a valuable review focused not only on liposomes, but on a larger range of nanocarriers, because all of them have a common set of shortcomings regardless of their chemical nature [76]. Essentially, they identified the following pitfalls of liposomes and nanocarriers in general: (i) overestimation of EPR effect, (ii) lower tumor and tissue penetration than expected, (iii) overestimation of active targeting; (iv) tendency to design complex materials without feasible applicability, (v) tumor biology is not considered in the drug formulation design, (vi) the formulations are designed rather for accumulation at the tumor site while metastases are not considered, (vii) the potential of nanocarriers in personalized therapy is not exploited accordingly, and (viii) inaccurate selection of an animal model (this aspect is discussed in the present review for GBM in Section 5). However, despite all these limitations, gaps, and misinterpretations, a number of nanomedicines, especially those based on liposomes succeeded to reach the market [32,33,35]. This success could be explained by the fact that benefits coming from the encapsulation of the drug in a carrier (e.g., liposome) exceeded the imperfections of nanocarrier-based formulations. It is henceforth feasible to continue the study on nanocarriers, particularly liposomal TMZ, for GBM treatment but keeping in mind the very complex nature of the biological system in which a new one, that is, the tumor and its environment, is growing. This is, however, a process requiring time, funding, and more important, patience, to properly understand the biocompatibility, biodistribution, selectivity, and efficacy of the designed TMZ liposomes. Another point of attention would be the safety of the designed liposomes. Being made of natural or natural identical phospholipids, liposomes themselves do not cause any harm to humans. In addition, the experiments of cell viability performed on uncoated liposomes in certain papers reviewed herein showed no or moderate toxicity, which encourage their use as safe nanocarreirs. Yet, to improve the efficacy of a liposome, it is not enough to prepare them from natural or natural identic phospholipids. The addition of a new function is prone to safety issues due to the possible toxic effects of the new component included in the liposomal formulation. Therefore, liposomes should be tested for each new formulation to properly address the safety issues that might appear as a result of change in chemical composition.

## 3. Preparation Techniques

### 3.1. Overview

**Figure 6:**
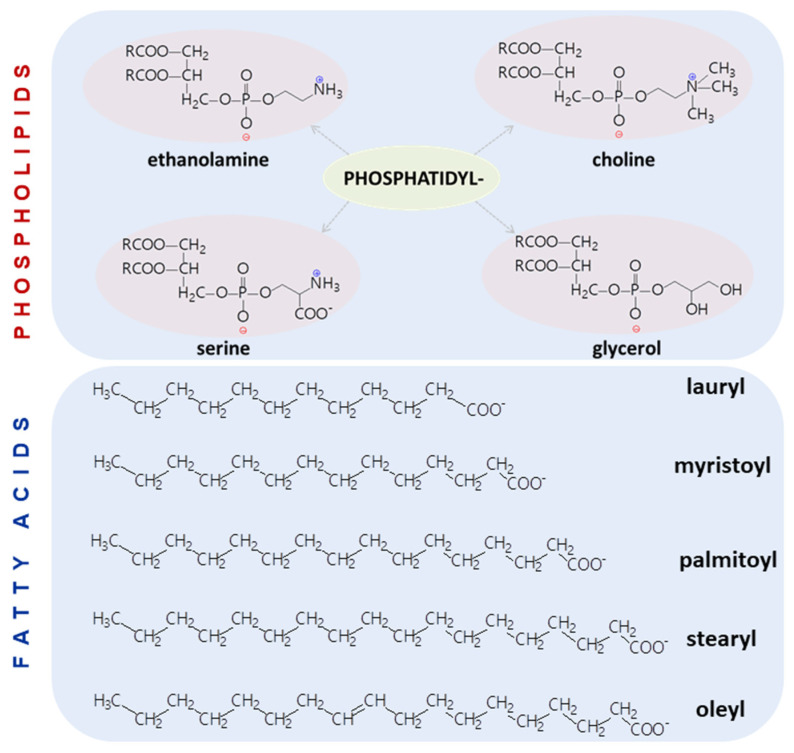
Common phospholipids used for liposomal formulations.

Chemical composition is the driving force of liposome formation and stability. So far, phospholipids of various origins, that is, natural, semi and fully synthetic, are being used for the preparation of liposomes. In addition, phospholipids modified from natural sources or phospholipids with natural head groups are also widely used in liposomal formulations [87,88]. Figure 6 illustrates the most commonly employed phospholipids in the preparation of liposomes.

Table 1 lists the main zwitterionic, anionic, and cationic phospholipids in liposome formulations, alongside their properties.

As can be noticed, the palette of lipids for liposome preparation is generous, and whatever lipid composition is used, organization into liposome-like architecture occurs. Because the physico-chemical properties of the phospholipids used in the preparation of liposomes greatly affect liposome properties and subsequent functionalization, the formulations of a liposome have to be performed by rationally selecting the lipids that will be organized in a particular liposome. For instance, the main influencing factor is the *transition temperature*, T_C_, which is the temperature needed to change the physical state of a lipid from an ordered gel phase (hydrocarbon chains are fully extended and closely packed) to a disordered liquid phase, where the hydrocarbon chains are randomly oriented and fluid [89]. This temperature depends on certain factors, such as hydrocarbon length, unsaturation, charge, and head-group substituent [87]. Indeed, the values provided in Table 1 for the selected (phospho)lipids vary considerably within the same group when the fatty acid moiety is changed. For example, increasing the number of carbon atoms from 12 to 18 changes the T_C_ value from −2 to 55 °C within the group of phophatidylcholines with saturated hydrocarbons chains. This can be explained by the stronger van der Waals interactions between the longer carbon chains, which require more energy to disturb from ordered packing. When a double bond exists in the carbon chain, the temperature at which the ordered packing arrangement occurs is lower (Table 1—oleyl-based phospholipids). Another critical parameter, *charge*, is related to the phospholipid head-group and influences liposome stability, pharmacokinetics, biodistribution, and cellular uptake [87]. The charge strongly controls the adsorption properties when the liposomes are in a real biological environment. For instance, the adsorption of proteins and other biochemical molecules, as well as the ability of liposomes to interact with cell membranes, which carry a net negative charge on their surface, is defined by the charge of the lipid components of the liposomes. The *long-term stability* of liposomes is of paramount importance for the conservation of a liposomal drug formulation. It is common knowledge that unsaturation is prone to oxidation. Therefore, a liposome made of phospholipids containing unsaturated fatty acids is expected to have a shorter life. Therefore, although the literature is rich in papers reporting liposomes prepared with phospholipids from biological sources (e.g., egg, bovine, or soybean), their translational level is quite low because such sources are rich in polyunsaturated fatty acids, which considerably reduces the long-term stability of the final product. On the other hand, synthetic phospholipids with higher T_C_ can raise difficulties in formulation due to the long and saturated hydrocarbon chains, which are more rigid and less permeable than shorter and unsaturated chains [87]. Under these conditions, a compromise would be to use a degree of unsaturation as low as possible if unsaturation is needed. To fulfill this requirement, the oleyl moiety is the most appropriate for liposomal formulation and in addition, due to their monounsaturated character, oleyl-based compounds exhibit much more stability as compared with polyunsaturated products. The stability of liposomal formulations is also strongly influenced by the degree of phospholipid hydrolysis in water [90]. The stability of liposomes under various conditions of pH, surface charge and ionic strength has been studied by Grit and Zuidam and the conclusion of their works are available in ref. [90].

As discussed above in Section 2, liposomes have different chemical compositions that influence their characteristics and ultimately their applications. Moreover, the methods used for liposome preparation greatly impact their properties and potential applications, including drug encapsulation rates and release kinetics, as well as the possibility of industrial manufacture and clinical use [15]. The criteria for choosing a particular preparation method for liposomes often include (i) the nature of materials used for liposomal synthesis; (ii) the nature and required concentration of encapsulated drug; (iii) the biological behavior of the formulation in relation to the targeted disease, as well as (iv) the clinical applicability and manufacturing scalability of the liposomal formulation [91].

**Figure 7:**
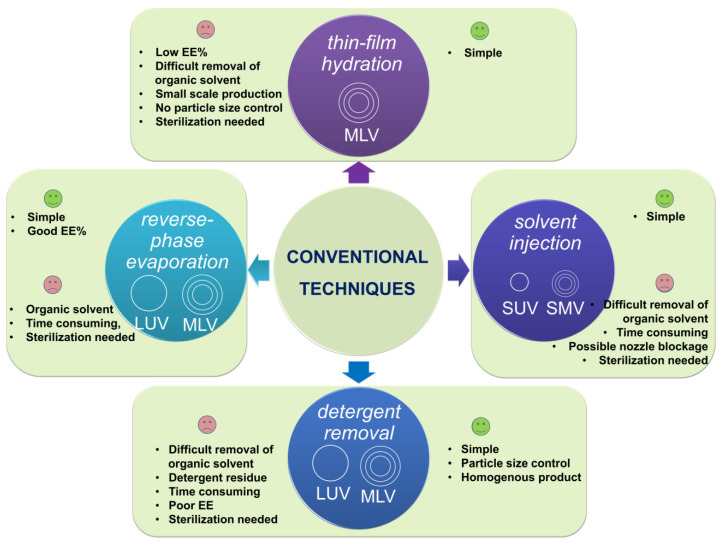
Main conventional techniques used for liposome preparation.

There ***are four main conventional techniques for liposome preparation*_,_** which have been mainly used for basic research purposes and proof-of-concept studies [92,93]. These methods differ mainly in terms of the way in which the lipids are dried down from the organic solvent and re-dispersed in aqueous media, and include *thin-film hydration*, *reverse-phase evaporation*, *solvent injection*, and *detergent depletion* [94,95,96]. Figure 7 illustrates the main conventional techniques used for liposome preparation alongside the principal benefits, shortcomings, and type of liposome with respect to size and structure (vide infra).

**Figure 8:**
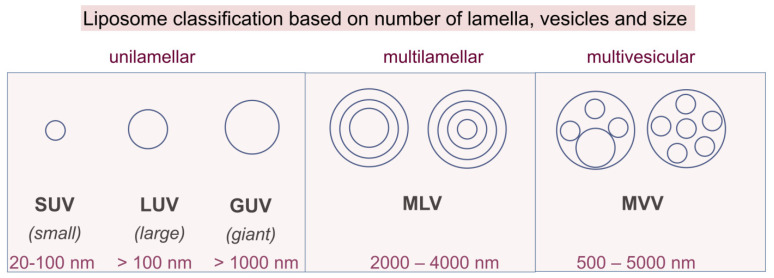
Types of liposomes based on structure and size.

Since they lead to the formation of a heterogeneous population of liposomes, conventional methods usually require *post-processing steps* for obtaining smaller vesicles or a more uniform size distribution, including homogenization, sonication, and extrusion [96]. The application of these batch preparation methods for clinically approved formulations has been, however, limited and problematic due the difficulty of generating GMP-compliant aseptic manufacturing methods. In 2011, for example, the European Medicines Agency (EMA) and the Food and Drug Administration (FDA) identified shortcomings in the quality management system of clinically approved doxorubucin liposomal formulation production (Doxil/Caelyx), which led to the permanent closure of the manufacturing site [97]. However, novel scalable approaches which permit a continuous manufacturing process, coupled with process analytical technology for maintaining strict control over the physical properties of the NPs, could help alleviate these shortcomings [97,98]. Depending on structure and size, liposomes can be classified into several groups, as depicted in Figure 8 [99,100,101]. The first classification refers to the structure, when three main types of liposomes can be found, i.e., unilamellar, multilamellar, and multivesicular. Then, the division of liposomes in subfamilies depends on size, which can be in the range of 20 nm to 5000 nm or even larger.

***Large-scale production techniques for liposomes*** include the heating method, freeze drying, spray drying, electroformation, the use of supercritical fluids, as well as several modified ethanol injection techniques which are increasingly attractive for the industry sector, including cross-flow injection and microfluidic mixing [102]. Examples of manufacturing process parameters which have been shown to influence liposome stability and drug encapsulation and release kinetics in large-scale production techniques are shear force, pressure, pH, temperature, lyophilization, as well as sterilization parameters [103], especially in the case of sterile filtration, where components can interact with the filtering membrane or matrix, which can in turn compromise the integrity and structure of the liposomes [104]. While the preparation, characterization and clinical applicability of liposomes have been described in detail elsewhere [91,105,106,107] a brief description of conventional and large-scale production methods for liposomes is given below, alongside with a short discussion over the existent production methods for TMZ-loaded lipid nanovehicles.

### 3.2. Conventional Methods

#### 3.2.1. Thin-Film Hydration (THF)

This is the most widely used method for liposome preparation and was the first to be described for liposome production [108]. Briefly, if the drug is lipophilic, a mixture of lipid material and drug is first dissolved in an organic solvent. Then, as the organic solvent is removed through evaporation, a thin lipid film is formed. The lipid film is subsequently hydrated with an aqueous solution under continuous stirring at a temperature higher than the lipid phase transition temperature to obtain drug-loaded liposomes. Hydrophilic drugs to be encapsulated should be dissolved in the aqueous solution used for film hydration. Although this method is easy and thus widespread in the scientific community, it yields a population of large and heterogeneous MLVs with low EE% [109]. It has additional drawbacks, such as the use of organic solvents, limited particle size control, and unsuitability for mass production [96]. However, sonication during hydration followed by liposome size reduction techniques, such as membrane extrusion, leads to the generation of homogeneous SUVs [110], which can be further functionalized for targeted liposomes generation. A more consistent size distribution upon hydration can be obtained by using sieved proliposomes generated through the film deposition on carrier method [111]. Here, the lipid components are mixed with a water-soluble carrier such as mannitol or sorbitol into an organic solvent, and the mixture is then dried down, ground and sieved, after which it is hydrated to form liposomes.

Since TMZ is an amphiphilic molecule, with a relatively high lipophilicity and slight solubility in water [31,112], knowing its exact distribution in the aqueous or lipid phase of liposomes is somehow challenging. In addition, only recently has it been demonstrated that TMZ’s partition is dependent on liposome lipid composition and pH [113]. For this reason, reports of TMZ-loaded liposomes generated through thin-film hydration use TMZ dissolved either in the lipid [51,55,82] or in the aqueous phase [52,80]. The reported TMZ-encapsulating MLVs are generally extruded for obtaining a more homogenous population of SUVs, although unextruded LUVs formed by hydrating sieved dry lipid films (proliposomes) have also been reported [9,114], as well as SUVs obtained through sonication of hydrated lyophilized lipid cakes [52].

Four types of TMZ-encapsulating cationic liposomes were generated by hydrating a TMZ and lipid-containing film formed by the slow evaporation of a mixture of 1:1 molar ratio TMZ:lipids [55]. The lipids used for this purpose were different combinations of DOTAP, Chol, DOPE, and DC-Chol. The mixture was dissolved in chloroform and methanol, placed on a rotary evaporator set at 65 °C for 4 h to produce the lipid film, which was then rehydrated in PBS, and extruded through a 0.1 µm polycarbonate filter. Bare TMZ-encapsulating liposomes had hydrodynamic diameters in the 100–150 nm range, and incubation of these liposomes with human plasma, which allowed for plasma protein adsorption, led to a size increase of 20–40 nm. The EE% of these formulations ranged from 41 to 54.9%, with a 16.6–22% drug loading content (reported to total liposome mass). Results showed that adsorbed plasma proteins facilitated a five-fold enhancement of antitumor activity of DOTAP/DOPE TMZ-encapsulating liposomes on U87 glioma cells when compared to their non-incubated analogues. This formulation was shown to preferentially adsorb plasma proteins which have a particular preference for receptors overexpressed in the BBB [55]. To confirm this, the authors also tested this formulation in an in vitro HUVEC BBB model, with results showing a high uptake of protein-decorated TMZ-encapsulating liposomes in this BBB model. However, HUVEC models are increasingly being recognized as unreliable BBB models [115], and further studies should be performed in vivo.

TMZ-loaded liposomes have been also generated by hydrating sieved proliposomes obtained through the film deposition on carrier method using mannitol [114] and sorbitol [9] as carriers, but with low EE% (22.68% and 35.45%, respectively). Interestingly, in the case of mannitol-stabilized proliposomes, TMZ was dissolved in the lipid phase, and liposomes were formed through hydration of the sieved proliposome powder with acetate buffer, whereas in the case of sorbitol-stabilized proliposomes, TMZ was dissolved in the hydrating solution. Yet, further investigations regarding TMZ intraliposomal distribution for improving EE% are necessary.

TMZ encapsulated in extruded *stealth liposomes* made of DPPC, Chol, DSPE-PEG2000, and α-tocopherol in a 3:1:1:0.004 molar ratio has also been reported [51]. TMZ and lipid components were dissolved in a mixture of methanol and chloroform and dried in a flask to produce a homogeneous lipid film, which was then hydrated with ammonium sulfate (pH 4.0) at 42 °C until it was fully dispersed to form liposomes. The suspension was then extruded successively through 200- and 100-nm polycarbonate filters. Subsequently, non-encapsulated TMZ was removed by passing the liposome-TMZ mixture through a spin column equilibrated with PBS (pH 7.0) to maintain the osmolality in biological media [51]. This formulation was shown to have an enhanced cytotoxic effect on U87 cells and a positive impact on survival time for mice bearing U87 xenografts when compared to both TMZ alone and controls. However, an augmented drug release pattern was observed for this liposomal formulation at 37 °C when compared to 25 or 4 °C, suggesting that this liposome formulation is not entirely stable at this temperature, which is close to the phase transition temperature of DPPC.

Several formulations for TMZ-encapsulating stealth liposomes were also generated by a modified TFH technique, which involved lipid solvation (DSPC, cholesterol, and DSPE-PEG2000) in tert-butanol, followed by overnight lyophilization [52]. The lyophilized mixture was hydrated with TMZ dissolved in citric acid buffer (pH = 3), and then rotated at 60 °C for 40 min. The obtained liposomes were homogenized on ice for 6 min. Unencapsulated TMZ was removed by incubation of liposomes with salicylic acid (SAC) at 4 °C, through the formation of SAC/TMZ co-crystals. It was found that the most successful formulation of TMZ-encapsulating liposomes in terms of encapsulation yield was composed of DSPC:Chol:DSPE-PEG2000 in a 10:8:1 molar ratio (up to 1.9 mg/mL loaded drug, with less than 20% of liposomal TMZ being unencapsulated). These liposomes could improve rat survival by 22% when compared to rats treated with TMZ alone. The authors highlighted that the pH-dependent stability of TMZ restricts the working pH in the liposomal preparation procedure, and that care should be taken when working with this small molecule, which spontaneously undergoes hydrolyzation to MTIC at physiological pH [52]. Polymers, such as chitosan, which can potentially improve the oral bioavailability of liposomes by organizing into a protective shell that can increase liposomal stability, especially in an acidic environment [116], have not been yet integrated in TMZ-encapsulating liposomal formulations.

A recent study reports the generation of transferrin-functionalized PEGylated DSPC:Cholesterol:POPG *targeted liposomes* loaded with a combination of TMZ and the bromodomain inhibitor JQ1 through TFH/sonication followed by extrusion [81]. A thin film of these materials was generated by rotary evaporation at 40 °C at 150 mbar and desiccated overnight until completely dry. Hydration of the lipid film was conducted at 65 °C under sonication in citrate buffer (pH 4) for 1 h. To reduce size and increase homogeneity, functionalized liposomes were filtered through a 0.2 μm PES syringe filter, and TMZ was finally added to the solution and loaded through a pH-gradient method. Treatment with these targeted liposomes prolonged survival and decreased tumor burden to a greater extent when compared to free drugs or drug-loaded non-functionalized PEGylated liposomes, suggesting that transferrin functionalization facilitates nanoparticle transport across the BBB for an improved antitumor effect.

In another study, through the insertion of a peptide recognizable by BBB transporters into the liposomal membrane, targeted TMZ-encapsulating liposomes showed a 35% increase in brain penetration and prolonged mouse survival in U87 xenograft-bearing mice [82]. The liposomes were prepared from cholesterol-ovine wool, l-α-phosphatidic acid sodium salt (egg, chicken), and l-α-phosphatidylcholine (egg, chicken) in chloroform. The targeting peptide, a short sequence of several amino acids (1,2-dioleoyl-sn-glycero-3-succinate-Cys-His-Leu-Asp-Ile-Ile-Trp-COOH) present in the amyloid precursor protein (APP), was dissolved in a methanol-chloroform solution and added to the lipid mixture. TMZ was added to the liposomes after dissolution in methanol. The solvent was removed by rotary evaporation at 40 °C for 15–20 min. The lyophilized lipid film was hydrated with phosphate buffer pH 7.4 and until complete dispersion. Liposomes were subjected to six freeze (liquid nitrogen) and thaw (37 °C hot bath) cycles, followed by extrusion through 200 and 100 nm polycarbonate filters. Liposomes were lyophilized overnight, and the dry liposomes were kept at 4 °C in sealed vials until use. Results showed that TMZ-encapsulating targeted liposomes had a 7.8-fold increase in mouse brain penetration and crossed the endothelial layer in a BBB model 3.5-fold more when compared to non-targeted liposomes with the same composition, demonstrating the successful enhancement of BBB permeability of liposomal TMZ. However, the authors did not calculate EE%, nor did they report means of eliminating non-encapsulated TMZ, making comparison with free TMZ difficult.

TMZ-liposomes composed of EPC:Chol:DSPE-PEG2000-Maleimide:DSPE-PEG2000 were specifically tailored for CD133^+^/ALDH1^+^glioblastoma stem cell uptake and BBB crossing through maleimide crosslinking to the thiolated forms of an anti-CD133 monoclonal antibody and angiopep-2 (An2), a LRP1 ligand which exhibits a better BBB penetration capability than other proteins, including transferrin [117]. Lipid components were dissolved in an organic phase (chloroform/MeOH) and deposited as a thin film, which was subsequently hydrated with 1 mL solution of TMZ in PB (pH 7.2). The liposome suspension was freeze-thawed for 10 cycles and extruded through double-stacked membrane filters of 450 and 100 nm, respectively, to generate liposomes which were then conjugated to CD133 mAb and An2 to form CD133 mAb/An2-conjugated immunoliposomes with a mean size of 203.4 nm and 99.2% encapsulation efficiency.

In the attempt of maximizing BBB penetration by liposomes carrying TMZ, Zhang et al. prepared glucose-decorated liposomes containing TMZ and PAP (TMZ: PAP, 6:4, *w*/*w*) through THF [83]. They mixed soy PC, cholesterol, and DSPE-PEG2000-MA (maltobionic acid) in a round-bottom flask containing a methanol/chloroform mixture. The solvents were removed with a rotary evaporator to leave a thin film in the flask. The film was dried overnight in a vacuumed desiccator and rehydrated with a PBS solution of TMZ and PAP to obtain the final liposomal formulation. The resulting liposomes were sonicated in an ice bath for 3 min and then extruded for a homogenous size distribution. The EE% for TMZ was 79.32%. This formulation was very efficient for liposomes to pass through the BBB and kill the tumor cells.

#### 3.2.2. Emulsification

Water-in-oil (W/O) emulsification followed by slow removal of the organic solvent has been extensively used for liposome production in a method known as reverse-phase evaporation (REV) [118]. This technique allows the entrapment of a large amount of the aqueous phase inside the liposome core, making it particularly suitable for hydrophilic drugs. First, the lipid components are dissolved in an organic solvent completely immiscible with water. The aqueous phase is then slowly added, and the mixture sonicated in order to produce reverse micelles. Slow removal of the organic solvent will lead to the generation of a viscous gel phase, which subsequently becomes an aqueous suspension of MLVs or LUVs [119]. The industrial applicability of this method is limited due to the high complexity of the manufacturing process, as well as the residual traces of organic solvents in the final product [94]. In addition, biologically active molecules, such as peptides, are not suitable for liposomal encapsulation using this technique due to the extended contact of the compound with the organic solvent [120]. Simple oil-in-water (O/W) emulsification can also be used for liposome formation, although this method requires high shear forces for reproducible liposome formation if no emulsifiers are used [121]. Slow organic solvent removal from phospholipid-stabilized W/O/W double emulsions can also lead to the generation of GUVs with good EE% [122].

The suitability of REV for generating TMZ-loaded liposomes has been attributed to the mild conditions used (room temperature), which are ideal for heat-labile compounds such as TMZ [2], even though the obligatory sonication step has a thermal effect on the sample. PEGylated TMZ-encapsulating liposomes generated through emulsification, followed by ultrasonic homogenization, showed a 54% encapsulation efficiency and average hydrodynamic diameter of 131 nm [80]. Briefly, an aqueous solution containing TMZ was slowly added under continuous stirring to a mixture of all lipid components dissolved in chloroform. MLVs were generated through organic solvent removal by continuous stirring at room temperature. Size and lamellarity reduction were achieved through sonication. In this case, TMZ was distributed in the aqueous phase due to its hydrophilicity, although it is unclear at what concentration was this drug used for encapsulation.

Simple oil-in-water (O/W) emulsification was also used for generating bare liposomes from egg PC and Chol, which were subsequently loaded with TMZ through the ammonium sulfate gradient method [28]. Briefly, an ethanol solution containing all lipid components was slowly poured into an ammonium sulfate aqueous solution at 60 °C, and after the ethanol was completely volatilized under continuous stirring, the mixture was sonicated. Dialysis was performed three times in normal saline, after which the bare liposomes were loaded with TMZ by incubation of the dialyzed suspension with an acidic solution of TMZ. Liposomal TMZ was separated from non-encapsulated TMZ through size exclusion chromatography, with a 90.3% entrapment efficiency for a heterogeneous population of liposomes with an average size of 185 nm [53]. Entrapment efficiency was found to be dependent on drug-to-lipid ratio, temperature, and concentration of initial ammonium sulfate solution.

#### 3.2.3. Ethanol Injection

Ethanol injection is one of the earliest methods employed for liposome preparation, being proposed by Batzri and Korn in 1973 as one of the first alternatives to thin-film hydration for the generation of uniform SUVs without sonication [123]. The method consists of injecting an ethanolic solution of phospholipids into an aqueous phase through a fine needle, followed by ultrafiltration. This leads to the spontaneous formation of SUVs or small MLVs, depending on the lipid components’ solubility in the organic phase. This method is simple and reproducible, and mostly suited for lipophilic drugs due to higher EE% [124], but usually requires organic solvent removal after liposome formation. Thin layer chromatography indicated no oxidative degradation of phospholipids during this procedure due to the protective atmosphere (i.e., N_2_ gas), whereas electron microscopy showed a reasonably homogeneous preparation of vesicles with an average diameter of about 26.5 nm. This method is fast, highly reproducible, easy to scale-up, and suitable for the entrapment of many different substances, such as large hydrophilic proteins or small amphiphilic drugs, and no additional sterilization step is needed [123,125,126].

A recent patent describes an ethanol injection method for preparing targeted TMZ cationic liposome complexes which can cross the BBB for the treatment of primary or metastatic brain tumors by intravenous administration [127]. Key steps in targeted liposome manufacture described in the patent include: (i) preparing an ethanolic solution of DOTAP and DOPE or a mixture of DDAB and DOPE with or without Chol; (ii) preparing a solution of TMZ in DMSO or another appropriate solvent, (iii) mixing the lipid and TMZ solutions; (iv) injecting the formed solution in aqueous media for the generation of cationic TMZ liposomes; (v) mixing the cationic TMZ liposomes with a proper ligand for targeted therapy. The ligand can be an antibody targeted to the transferrin receptor, folate receptor, or HER-2 receptor, an antibody fragment or protein. This leads to the generation of a targeted TMZ cationic liposome in which the ligand is not chemically conjugated to the TMZ-loaded liposome. A tumor-targeting immunoliposome nanocomplex decorated with an anti-transferrin receptor single-chain antibody fragment (“scL-TMZ”) prepared using this method has been shown to possess an enhanced anti-cancer effect in animal models of GBM when compared to free TMZ [78]. The size of the scL-TMZ was 41.4 ± 9.2 nm, while the EE% of TMZ was 45.23 ± 4.34%. Chemical conjugation between drug-loaded liposomes and the ligand for targeted therapy is also possible through the inclusion of up to 5% maleimidophenylbutyrate-DOPE (MPB-DOPE) in the lipid formulation, provided that the ligand contains cysteine residues which can be reduced to sulfhydryl for maleimide crosslinking to MPB-DOPE [128].

Following reports of a synergistic effect of quercetin (QUE) and TMZ on in vitro tumor models, polymeric nanoliposomes encapsulating both TMZ and QUE were generated by slowly injecting a mixture composed of soy lecithin, QUE and TMZ in ethanol/acetone, melted cholesterol and glyceryl behenate, into an aqueous solution containing a suspension of DSPE-PEG2000 in Poloxamer 188 and Tween-20, under continuous stirring [47]. The authors propose that this formulation is composed of a lipid core which contains all hydrophobic components and the two drugs, and is surrounded by an aqueous bilayer formed by components of the aqueous phase, that is, Poloxamer 188, Tween-20, and DSPE-PEG2000. The reported size of these polymeric nanoliposomes was 100–300 nm while the EE% of TMZ ranged from 53.58 to 66.35%. This nanoformulation of QUE/TMZ exhibited a 1.8-fold increase in cytotoxicity when compared to free TMZ on U87 glioma cells, as well as good activity on TMZ-resistant cells, and biodistribution studies in a glioma rat model showed a significant accumulation of the QUE/TMZ-loaded polymeric liposomes in the brain and liver [47].

One study found that the ethanol (or isopropanol) injection method was not suitable for generating TMZ-encapsulating liposomes due to the instability of TMZ in ethanol, as demonstrated by the presence of its hydrolysis product, MTIC, in an ethanolic solution of TMZ [52]. This suggests that this method is not suitable for TMZ encapsulation if the non-hydrolyzed form is desired in the final liposomal product.

#### 3.2.4. Detergent Removal

In this method, mixed micelles are formed upon lipid solubilization with a detergent at critical micelle concentration (CMC), followed by dialysis or column chromatography for detergent removal and liposome self-assembly [129]. This method usually leads to the formation of a homogenous population of unilamellar liposomes and has been shown to be suitable for a wide variety of polar lipids, with particle size being dependent on the surfactant, the surfactant-to-lipid ratio, and rate of detergent removal [130]. However, this method is time consuming, leads to a low concentration of liposomal vesicles in the final product, which can also contain residual surfactant, making it unsuitable for up scaling due to difficult process control [104]. No TMZ liposomes have been generated through this method to our knowledge.

### 3.3. Large-Scale Liposome Production Techniques

**Figure 9:**
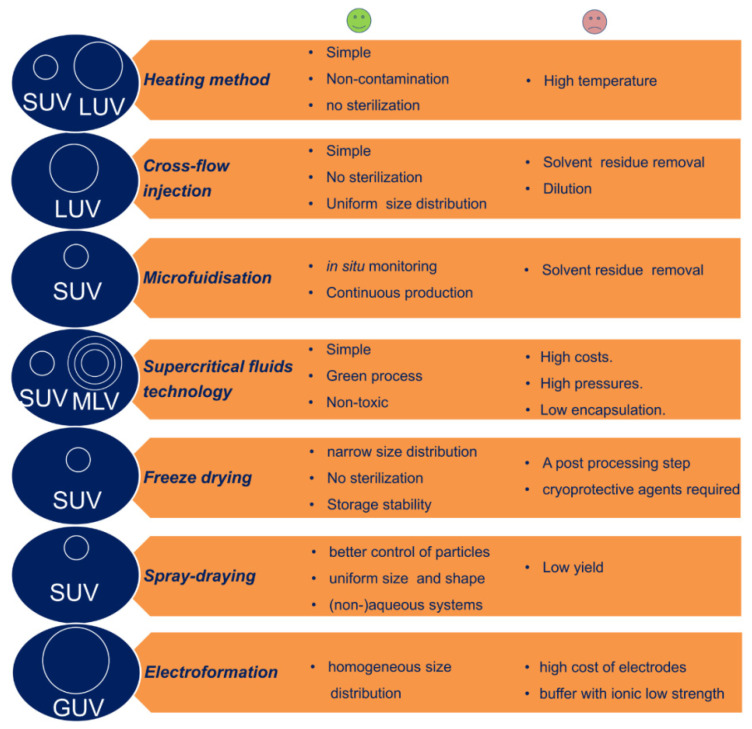
Main methods for large-scale production of liposomes.

Although the large-scale production of liposomes has increasingly penetrated the market starting from last year, it is still a subject in its infancy, a fact which opens up a new avenue in the development of these carriers with profound implications in the health and economic sectors. According to the Transparence Market Research, the Pharma industry sector focused on liposome drug delivery has accelerated research and development activities for novel liposomal formulations of drugs and vaccines [131]. Hence, after an estimated revenue of US$ 3.6 Bn in 2018, the liposome drug delivery market is expected to experience a favorable year after year growth, with an estimated revenue of ~US$ 8 Bn by the end of 2027. Yet, the unprecedented production of liposomes lately raises several questions regarding the methods used for manufacturing, especially since the literature is abundant in discussions over a large variety of methods, each of them having both advantages and drawbacks [104,109,132,133,134,135,136]. To obtain the final marketable product, there are several production steps to follow, that is, mixing, separation, hydration, homogenization, sterilization, and stabilization [134]. First, several formulations are prepared, subjected to physico-chemical characterization, followed by extensive in vitro and in vivo studies from which a “leading” formulation is selected for large-scale translation. Yet, this step can be very challenging, especially when a conventional technique, such as thin film hydration, is used, followed by a down-scaling process, that is, extrusion or sonication. Problems which arise are often related to batch to batch variation, difficulty to scale up, stability, reproducible in vitro release data etc., reinforcing the idea that a universal preparative method is challenging to provide. However, the FDA released a document providing guidelines for the industrial manufacture of liposomes, which focuses on technical aspects of liposomal drug formulations irrespective of the selected method [135]. Besides, the additional steps mentioned above for manufacturing could considerably increase the price of final product. An overview on the methods used or with a high potential for translation at an industrial scale provides a clear picture on the currently existing methods for liposome manufacturing. Figure 9 displays the main methods used for liposome large-scale production or with high scale-up potential, along with the principal advantages, disadvantages, and types of obtained liposomes.

To the best of our knowledge, none of these methods has yet been used for the large-scale preparation of liposomal TMZ. As one of the scopes of this review is to identify the levels reached by basic and translational research in the development of liposomal TMZ for GBM, detailing these methods is beyond the goal of this work and thus, they will not be described herein. Yet, the interest reader can find consistent information regarding this subject in references [95,96,102,136,137,138,139,140,141,142,143,144,145,146,147,148,149,150,151,152,153,154,155,156,157,158,159,160,161,162,163,164,165,166,167].

## 4. Physico-Chemical Properties

**Figure 10:**
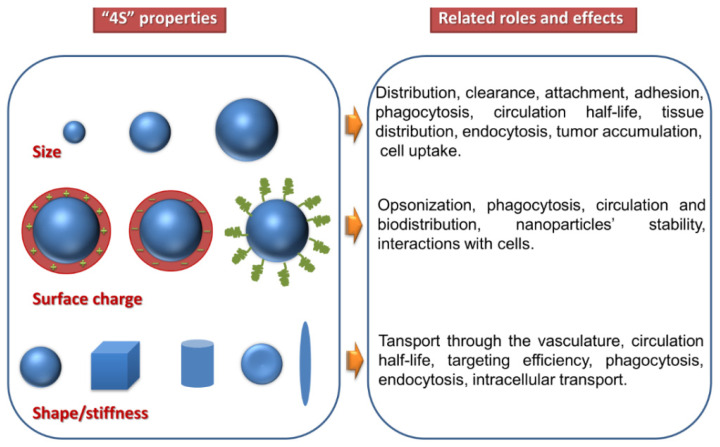
Physico-chemical properties (4S) influencing the fate of NPs after intravenous administration.

Once intravenously administered, liposomes, as well as NPs in general, should circulate through the blood stream enough time to reach the targeting tissue and accumulate into the cell cytoplasm. Yet, this desiderate is strongly dependent on the physico-chemical properties of the NPs, the so-called “4S“ parameters (i.e., Size, Shape, Stiffness, and Surface chemistry) [168]. Figure 10 illustrates the critical parameters of NPs alongside the main roles played in their fate in the body.

**Figure 11:**
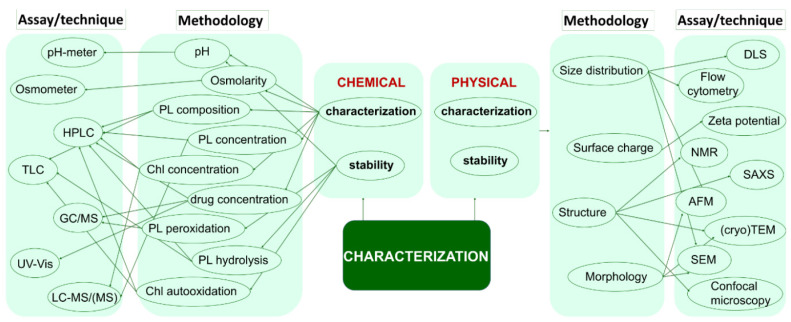
Physico-chemical characterization of liposomes: methodologies and assays/techniques.

As can be noticed, the characteristics of NPs influence their fate to an extremely high extent, starting from the point of administration to the point of tumor cell accumulation, where they should be able to effectively deliver the encapsulated chemotherapeutic agent. It can be stated that insights into the physico-chemical properties of NPs and the intermolecular interactions between these NPs and the biological environment are the main factors which guarantee the successful design of biocompatible smart nanostructures able to effectively deliver drugs into tumor cells. However, targeting theranostic NPs carrying a drug, a contrast agent, and a targeting molecule are even more complex structures exhibiting numerous physico-chemical parameters for which careful and profound evaluation is needed. Characterization methods have to be reproducible and precise for a fast translational process from bench to bedside. It is already acknowledged that the lack or scarce characterization of the NPs used for medical applications is considered to be the main reason for the failure of nanotechnology-based therapies. Therefore, a detailed characterization of the prepared liposomes/NPs is critical, in order to improve biodistribution, toxicity, clearance, and finally therapeutic efficiency of the delivered drug. More insightful discussions detailing how the properties of NPs can influence the body’s reactions to them and then their trafficking outside and inside cells, as well as the ability to specifically recognize cells and selectively discharge their payload can be found in several reviews [168,169,170]. Since the current review is mainly focused on development of liposomes carrying TMZ for brain cancer, the following discussion will be based on the properties of these prepared vehicles in relation to the biological effects observed either on cells or on animals, as appropriate. The physico-chemical properties of liposomes, which define their behavior in a biological environment, alongside the main characterization techniques, are illustrated in Figure 11 [43,170,171]. The list is not exhaustive, but covers the main characteristics and the corresponding techniques used to properly assess liposomes, aiming to prepare reproducible samples with high potential for translation toward the clinic.

The chemical assessment of liposomal drugs is made immediately after preparation, but also at regular periods to evaluate their stability over time. Usually, the type and concentration of phospholipids, their hydrolytic and oxidative products, as well the pH and oxidation products of cholesterol are the key parameters monitored to evaluate the chemical composition and long-term stability of liposomes. They are assessed by using common analytical methods, such as HPLC, TLC, GC, GC-MS, LC-MS, LC-MS/MS, UV-Vis etc., (Figure 11). On the other hand, the structural formation (mono-, bi- and multilayered liposomes), morphology, surface charge, size and size distribution are the main physical characteristics considered to confirm the successful preparation, reproducibility, and long-term stability of liposomes. The corresponding parameters are determined using specific techniques for the characterization of NPs, that is, DLS, zeta potential, optical microscopy, SEM, (cryo)TEM, AFM, confocal microscopy etc. The structure or lamellarity of liposomes is usually assessed by employing ^31^P-NMR, ^1^H-NMR, SAX, (cryo)TEM, and confocal microscopy, and it affects the EE%, the release kinetics of the encapsulated drug, as well as the fate of the drug in the body [28].

The results obtained so far for liposomal formulation of TMZ in terms of physico-chemical properties are vital because they provide a larger picture on what actually happens in biological media either in vitro or in vivo. Based on the observed behavior, these properties can be fine-tuned in strict relation with the final aim. For instance, Kim et al. developed a dual-targeting immunoliposome encapsulating TMZ by using angiopep-2 (for targeting the BBB) and anti-CD133 monoclonal antibody (for targeting the GBM stem cells) [80]. Size distribution and zeta potential of liposomes were measured by DLS. The authors characterized the freshly prepared liposomal formulations in relation to the size distribution, surface charge, and drug concentration. The size, zeta potential, and drug EE% were 203.4 nm, −1.6 mV, and 99.2%, respectively. Yet, no analysis of the two functionalities loaded on the liposome external surface was performed, which raises questions regarding the real amount of these two ligands in the samples. The results obtained on U87MG-TL and glioma stem cells reinforce the idea that the physico-chemical properties of the prepared liposomes, although partially assessed, were appropriate for both recognizing the tumor cells and enhanced local delivery of TMZ. Yet, the lack of including liposomes with varying physico-chemical properties does not allow for a proper estimation of the maximum biological effects on the investigated cell lines.

In another study, Lin et al. prepared a TMZ liposomal formulation (LipoTMZ), which has been characterized for the physical properties, that is, size, surface charge, and morphology by DLS and TEM, respectively. The neutral sphere-shaped liposomes had a size of 160 nm. HPLC was employed to quantify the amount of TMZ encapsulated in liposomes, providing an entrapment efficiency of 87% and a load of approximately 8.7 mg TMZ per mL of LipoTMZ suspension [51]. U87MG were treated with the obtained liposomes and after that, they were administrated to tumor-bearing mice by CED. In the first case, they bind to the cell surface and then are internalized into the nucleus, proving that liposomes have adequate properties for cellular uptake and nuclear distribution. The delivery of LipoTMZ to the brain by CED resulted in a significantly prolonged survival of tumor-bearing mice as compared with free TMZ and liposomes. These results showed that the liposomes exhibited suitable properties in terms of size and morphology to achieve a therapeutic effect.

Kong et al. prepared TMZ-liposomes for administration through nasal mucous membrane [28]. The samples were characterized for particle size and size distribution using a Beckman laser particle-size analyzer. A large distribution between 40 and ~500 nm with an average of 185 nm was obtained for the sphere-like liposomes, according to DLS and TEM, respectively. The authors noticed a close relationship between the amount of drug and lipids used for liposome production. Hence, when the drug-to-lipid ratio was 1:2, 1:4, and 1:6, the entrapping efficiency of TMZ-liposomes was 28, 62, and 90%, respectively, pointing out that higher drug-to-lipid ratios are more favorable in terms of entrapping efficiency. However, the lack of in vitro and in vivo tests does not allow for an analysis with respect to the biological effects.

Gao et al. formulated TMZ-liposomes from proliposomes and then investigated their physico-chemical properties, i.e., size, pH, morphology, followed by an evaluation of the amount of encapsulated TMZ [9]. The mean size of the sphere-like liposomes was 156.7 ± 11.4 nm with a narrow polydispersity index of 0.29 ± 0.04. The average drug entrapment efficiency and loading capacity, calculated based on HPLC, were 35.45 ± 1.48% and 2.81 ± 0.20%, respectively. The mean pH value of TMZ-liposomes was 6.46 ± 0.08, which was within the safe pH range of 4–9 for intravenous injection. The results of pharmacokinetic studies and tissue distribution showed that the liposomes had a slow-release and brain-targeting effect. Furthermore, TMZ-liposomes could reduce the C_max_ in some organs, such as heart and lung, potentially decreasing the side effects of free TMZ, indicating an improved biodistribution while the drug concentration at the target site was increased. Yet, the presence of sorbitol on the surface of the liposomes, which formed a hydrophilic layer, favored the accumulation of liposomes in the kidneys of tested animals. It can be stated that although the performance was not very high when compared with that of the free TMZ solution (Figure 6 in ref. [9]) the liposomes exhibited good properties, including activity at the glioma cells.

Nordling-David et al. developed a series of liposomes with different liposomal compositions, carrying either TMZ or Gd-DTPA for therapy and diagnosis, respectively [52]. All samples were first characterized by DLS to determine the size and zeta potential, then assessed for encapsulation yield (HPLC) and lipid concentration (HPLC). This screening resulted in an optimal liposomal formulation for TMZ with an average size of 121 nm and −0.2 mV zeta potential, which contained 28 mg/mL lipids and exhibited a 23% encapsulation efficiency. In addition, 63% of the total TMZ was preserved intact inside the liposome even after 1.5 years. This sample consisted of spherical unilamellar liposomes, according to cryoTEM results. The viability study on CNS-1 rat glioma cells indicated that treatment with TMZ-liposomes induced both a time- and a concentration-dependent decrease in the number of cells. At higher concentrations (1 and 2 mM), treatment with either TMZ-liposomes or free TMZ had a comparable effect (90% inhibition) indicating a successful controlled release of TMZ from the liposomes. According to the sustained release profile, liposomal TMZ was less effective than free TMZ, but the increased activity over time observed for the liposomal formulation positively influenced its in vivo behavior. It was noticed that animals treated with TMZ-liposomes survived ~22% longer than those treated with free TMZ. Moreover, the rats treated with TMZ-liposomes had significantly lower edema volumes seven days after treatment in comparison to free TMZ-treated animals. Although this study provides a systematic investigation on the physico-chemical characterization of liposomes exhibiting promising therapeutic effects, the presented in vivo results are not very conclusive about the effectiveness of CED for liposome delivery when compared to free drug solution.

Lam et al. developed transferrin-functionalized nanoliposomes for co-delivery of TMZ and bromodomain inhibitor JQ1 [81]. The resulted liposomes were characterized with respect to size, surface charge, and drug loading degree by DLS and HPLC. The average diameter of these liposomes was 137 nm with a zeta potential of −12.1 mV, whereas the EE% was relatively low (38.3% JQ1 and 45.1% TMZ) and overall drug quantities per total nanoparticle mass was very low (2.3% JQ1 and 2.5% TMZ). The brain uptake percent of particles with these properties was higher when compared with other targeted liposomes used in the work, that is, 1.7% of the injected dose. Therefore, liposomes carrying TMZ and having such properties were able to successfully cross the BBB of NCR nude mice. Moreover, in mice bearing U87MG and GL261 glioma cells, the tumor decrease was of 99.1 and 99.3%, respectively, after seven days of treatment, proving that the developed liposomes have the suitable properties to cross the BBB, target the tumor cells, and induce a therapeutic response.

In the study of Gabay et al., a drug-delivery system specially designed for transporting anti-cancer compounds (e.g., TMZ) via the BBB is presented [82]. The idea was to limit the exposure of peripheral organs to the drug, as well as the drug’s enzymatic degradation. Size, zeta potential, and polydispersity index of 137.4 nm, −49.9 mV, and PDI 0.083, respectively, of the liposomes were the only physical properties evaluated in this study. This work pointed out the superiority of the brain-targeted liposomal delivery vehicle possessing the above-mentioned physical parameters for the treatment of GBM, particularly in a SCID mouse model, as compared with the free drug.

Vanza et al. reported PEGylated liposomes that were optimized by using design of experiment (DOE) to find the effect of variable parameters (lipid ratio and organic phase ratio) on response parameters (%EE and % drug loading) at three levels (−1: low, 0: medium, +1: high) [2]. The particle size of the optimized TMZ-liposomal formulation (TMZ-PL) was 135.6 nm while the PDI value was found to be 0.202 ± 0.024, indicating a homogenous size distribution of the optimized formulation. The zeta potential of optimized TMZ-PL was found to be −26.26 ± 0.40 mV and related to the negatively charged lipid DPPG. The cryoTEM image of optimized TMZ-PL displayed unilamellar vesicles with spherical shape (Figure 4 in ref. [2]). The chemical characterization was focused on the drug content, specifically EE%, with a value of 56.11% for the optimized formulation. The authors also noticed an increase in EE% with increasing lipid molar concentration. An in vivo pharmacokinetic study was performed for the optimized liposomal formulation and free drug, which were intravenously administrated to rats. A higher concentration of the drug was measured in the brain upon delivery in PEGylated liposomes, as compared to the free drug solution (Figure 11 in ref. [2]). These outcomes show that the physico-chemical properties of the optimized formulation were adequate to avoid clearance by the immune system for successfully targeting the tissue of interest, the brain.

Kim et al. developed a “dual-targeting” immunoliposome nanocomplex carrying TMZ and decorated with anti-transferrin receptor single-chain antibody fragments to facilitate crossing of the BBB [78]. Liposomes were characterized for their size and surface charge by DLS, and the values of 41.4 ± 9.2 nm and 30.1 ± 4.6 mV for mean size and zeta potential, respectively, were obtained. The EE% of TMZ was of 45.23 ± 4.34%. Results revealed that liposomes with these characteristics were capable of killing cultured GBM cells much more effectively than free TMZ, confirming the ability of the liposomes to enter cells via the surface anti-transferrin receptor and to deliver TMZ in the intracytoplasmic compartment. However, non-GBM cells were also more sensitive to TMZ-liposomes than to free TMZ. Liposomes exhibiting these physico-chemical properties were capable of reaching normal endothelial cells of the BBB, as well as tumor cells in the brain in the employed experimental animals.

In order to improve efficacy of TMZ drug delivery to brain tumors, Hu et al. developed a DSPE-PEG2000 polymeric liposome in which quercetin (QUE)-TMZ (1:1 *w*/*w*) was co-incorporated [47]. The QUE/TMZ liposomal formulations were made of particles with a spherical shape according to TEM, with a narrow size distribution and an average of 196.5 ± 47.3 nm and a zeta potential of 30.5 ± 6.9 eV, as measured by a laser particle analyzer. The authors characterized the obtained formulations by UV-Vis, HPLC, and FT-IR, aiming to investigate the chemical composition, drug-carrier interaction, and drug distribution in the liposomal compartments. UV-Vis results showed that the core of the liposomes was not affected by the inclusion of TMZ. HPLC revealed that the QUE/TMZ ratio was not altered by the attachment of DSPE-PEG2000 while FT-IR measurements indicated no significant chemical interaction between drugs and the carrier. In addition, the two drugs, TMZ and QUE, were distributed in the middle of the lipid core, surrounded by the aqueous bilayer. These liposomes were able to encapsulate TMZ in the range of 53.58–66.25%, which did not affect the EE% of QUE, which was stable in the range of 69.42–78.37%. The authors noticed that QUE/TMZ liposomal formulations were more soluble in water, prolonged the circulation times of QUE in the blood, and exerted enhanced antitumor effects by killing both drug-sensitive and drug-resistant glioma cells due to the high intracellular drug concentration. The formulation proved to be more effective at killing glioma cells than free TMZ or QUE. The formulation induced morphological changes and apoptotic cell death at 100 µM following 24 h of incubation with the U87/TR cells. Biodistribution upon intraperitoneal injection in rats indicated that although the QUE/TMZ liposomes reached the tissues, they primarily accumulated in the brain and the liver [47].

The liposomes decorated with glucose prepared by Zhang et al. were characterized in view of their size, zeta potential, and morphology by DLS and TEM [83]. The obtained values of hydrodynamic diameter and zeta potential for the sphere-like liposomes were 133 nm (with PDI of 0.246) and −5.68 mV, respectively. To evaluate the stability of the prepared liposomes in cell culture medium and blood, the CMC of liposomes was also measured, and a value of 0.43 µg/mL was obtained. The stability over time of liposomes was assessed by size and PDI. The results obtained in vitro and in vivo revealed that the physico-chemical properties of the prepared liposomes were favorable for penetrating the GL261 cells, for reaching lysosomes and mitochondria, and finally for suppressing tumor progression. It was shown that the formulation was also able to penetrate the in vitro BBB model through the glucose-GLUT1 pathway. Nanosized liposomes with a negative surface decorated with glucose were preferentially accumulated at the tumor site when administrated intravenously to GL261-bearing mice, as compared with the liposomes without glucose, clearly highlighting the key role of glucose in passing the BBB. Liposomal formulations with the discussed properties were capable of shrinking the tumor while extending the life of mice up to several days as compared with those treated with free TMZ and glucose-nonmodified liposomes.

Table 2 summarizes the physico-chemical properties of the main TMZ liposomal formulations discussed in this review, the techniques used for characterization, as well as the in vitro and in vivo setups.

If the information provided in this table is critically analyzed in relation to the large number of techniques available to comprehensively characterize liposomes in view of technology transfer (Figure 11), it becomes obvious that liposomes prepared for the delivery of TMZ for brain cancer treatment generally lack an in-depth understanding in terms of physico-chemical properties influencing activity. In addition, assessment of the long-term stability of liposomal formulations of TMZ is reported even less often. The results presented above suggest the fact that most of the time, authors aim simply to observe what the prepared liposomal formulations of the drug are capable of doing in vitro and in vivo when compared with the free drug. However, such an approach is a key pitfall in liposomal design because the reproducibility of the liposomal formulations for brain targeting, as well as for tumor targeting in general, can be achieved only in accordance with key properties of the liposomes, which are obtained only by performing a systematic characterization of the formulations. Moreover, even if the costs for variation in formulation can seem high, if the experimental expenses for the failed in vitro and even more for the in vivo experiments are considered, we become aware of the large budget needed to prepare liposomes “in blind”, which are primarily tested for their biological effects on cells and animals instead of being properly characterized. One of the main consequences is the fact that it is difficult to obtain similar results when something is changed in the chemical formulation or even in the biological environment. Therefore, an in-depth characterization of drug-based liposomes before thoroughly investigating their ability to reach a target cell, to bind and enter the cells, and finally to discharge the payload would be a more economical approach and with increased chances to be transferred from bench to bedside.

## 5. Translational Oncology

In order to have a more precise estimation of the clinical outcome of novel therapeutic strategies, in this case, liposomal formulations of TMZ, intensive studies on lab animals bearing glioma models are required. Yet, in order to obtain a maximum effect of reproducibility of the results, tumors that are genetically similar to human GBM have to be used for the translational studies. So far, different approaches have been explored for developing glioma models in animals, that is, chemically induced models, xenograft transplantation models, and genetically engineered models [172,173]. These models have provided important insights into specific mechanisms of tumor initiation and progression, however, none of them can fully reflect the human gliomas, which results in a low degree of translation into more effective therapies [172]. As will be discussed below in Section 6, most of the studies carried out on free TMZ usually failed in phase 3. Although model GBM tumors have many limitations, they proved to be critical for the development of new drug formulations with high potential for enhanced clinical effects. Interestingly, apart from other animal models (e.g., rat, murine, canine, feline), dogs are prone to spontaneously developing brain tumors at any age, in any breed, and with no reported sex biases, whose incidence and malignancy exhibit similar features with human tumors [174]. Therefore, they provide a more realistic picture of the efficacy of drugs against GBM, opening up new avenues for the evaluation of novel nanotherapeutics. Because it falls out of the scope of this review, we will not present a deeper discussion on this subject, but the interested reader is invited to consult a recently published insightful review dealing with the employment of healthy and tumor-bearing dogs (most of them with brain tumors) aiming to understand (nano)drug pharmacokinetics and pharmacodynamics, mainly focused on human health [175]. It is however worth emphasizing that the use of dogs as models for human brain tumors is continuously expanding, especially in the last years, with the clear goal of assessing the effects of developed nanocarriers, having thus an enormous translational value and a high potential for clinical trial transition [176,177,178]. Yet, studies regarding liposomal nanocarriers for brain cancer delivery on such animal models are rather scarce. For instance, Dickinson et al. developed cationic liposomes containing topoisomerase inhibitor CPT-11 that were delivered by CED to a canine spontaneously generated brain tumor, whose volume was monitored by MRI and histopathology [179]. They noticed that poor infusions with early leakage and poor infusate distribution volumes (Vd) caused insignificant changes in tumor volume or imaging characteristics, while an increased Vd and percent coverage of the tumor volume resulted in an apparent reduction of tumor volume, necrosis, and decreased or static tumor growth. Bredlau et al. prepared temperature sensitive liposomes that were intravenously administrated to dogs [180]. The aim was to evaluate the ability to cross the BBB, as well as the fast release of the contained drug in response to hyperthermia (>40 °C). These studies are very important because they provide valuable information on the optimization of liposomal-based therapies for brain cancer in order to move to the next level, that is, human glioma clinical trials, and thus to push the frontiers of evidence-based knowledge of this lethal disease.

Concerning the delivery of TMZ encapsulated in a nanocarrier to canine spontaneous brain tumors, only one report can be found in the literature, to the best of our knowledge [181]. The authors synthesized theranostic NPs by incorporating TMZ in polymeric magnetite carriers against brain tumors spontaneously developed by dogs. The nanotheranostics were administrated by CED, followed by MRI evaluation in relation to the tumor evolution. Despite the limitations of the study, that is, short infusion time, constant volume of infusate, lack of serial follow-up MRI, and only achieving 70% target accuracy, this is the first study showing the feasibility of delivering TMZ nanopolymeric formulations in canine brain cancer and the second one employing CED as an efficient technique of administration.

## 6. Clinical Trials

**Figure 12:**
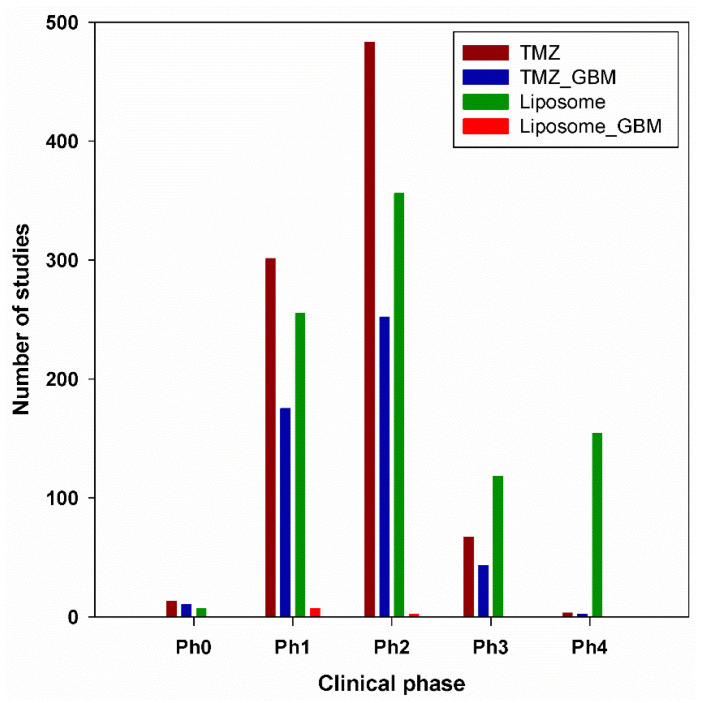
Clinical trials on TMZ and liposomes.

Despite the developmental challenges of nanomedicines for glioma/GBM treatment, liposome-based formulations have already occupied an important place in clinical trials. Hence, the first clinical trial (phase 1) using liposomes started in 1999 and was focused on liposomal doxorubicin for pediatric patients with refractory solid tumors [182]. The most recent phase 1/phase 2 trial started in 2020 with the purpose of studying the side effects and the best dose of liposomal verteporfin, as well as investigate its efficiency in treating patients with high grade recurrent EGFR-mutated GBM [182]. However, as can be seen in Figure 12, only a few clinical trials using liposomes to target GBM have been conducted, and none of them with TMZ as the therapeutic agent.

Essentially, it can be noticed that free TMZ and liposomes carrying a wide variety of drugs have been approved for a large number of clinical trials (hundreds of studies).

Even if the most clinical trials for free TMZ are limited to phase 1 and phase 2, their large number indicates a much more interest for this drug as compared with other drugs encapsulated in liposomes. On the other hand, the complexity of the nanoformulations, requiring more in-depth physico-chemical characterization (e.g., size, surface charge, chemical composition etc.,), as well as information on the drug loading degree, pharmacokinetics, biodistribution, cell internalization, and biological effects, could slower the process for clinical trial approval. All things considered, the number of clinical trials on liposomal formulations is quite impressive. Moreover, the number of clinical trials reaching phase 3 and even phase 4, which are for liposomal drugs authorized by the FDA to have reached the general population due to the proven safety, efficacy, and quality [183], is quite high, holding a great promise for their use for TMZ encapsulation. This is particularly expected since the clinical trials show that only a limited number of studies on free TMZ were able to reach phase 3 and even fewer reaching phase 4. On the basis of the benefits brought by liposomes, it is therefore expected that the encapsulation of TMZ into such carriers will overcome the current shortcomings of free TMZ-based therapy and significantly improve the treatment efficiency, reduce systemic toxicity, and ultimately enhance the survival rate. These are solid premises for technology transfer [81].

It is worth mentioning that in most cases, the peculiarities of brain neoplasia and TMZ chemical conversion in systemic circulation [8] stimulate the incorporation of TMZ into liposomes in the attempt to defining an optimal drug delivery system for this treatment. Yet, none of these studies could reach any of the clinical trial phases, showing a low translational process from bench to bedside. Experimentally, a translational effect is expected to occur only when the tests meet a comprehensive investigation of all aspects that need to be interconnected to generate a therapeutic prescription, that is, GBM morphopathology, TMZ as chemotherapeutic agent and its pharmacokinetics, liposome composition and size, carrier interaction with biological fluids, accessing and overcoming the BBB, penetration inside tumor, and a maximized therapeutic effect. As discussed above in the translational studies section (Section 5), the scientific community is making great efforts, particularly in the last years, for identifying the most appropriate and realistic models for human GBM, with the clear goal of filling the gap between these two critical steps—animal toward human studies. So far, the average rate of successful translation from animal models to clinical cancer trials is less than 8% [184]. The new outcomes from studies on dogs are promising, since they offer an increasing body of evidence as proof-of-concept data to advance their application in humans [185], yet reports on liposome-based NPs with TMZ targeting canine intracranial tumors are still far of being addressed to a greater extent.

## 7. Conclusions and Perspectives

The current review summarizes the present-day efforts of the scientific community for maximizing the therapeutic effect of TMZ by encapsulation in liposomes as biocompatible (nano)carriers, with the aim of targeting GBM, a deadly disease. The scope of this work was to emphasize the challenges and the opportunities associated with this research area aiming to develop promising TMZ formulations for the effective treatment of GBM. Despite of all the available literature and efforts made in liposomal TMZ delivery systems, there is still a great need for novel formulations with high potential for clinical translation. The literature reviewed herein revealed many approaches to preparing liposomal formulations of TMZ, each of them having benefits and shortcomings. It is important to note that the methods which have been used so far for producing liposomal TMZ are still at a basic research level and have not yet been scaled-up for industrial manufacture. Moreover, the protection of TMZ through liposomal encapsulation proved to be an efficient strategy that avoids the hydrolysis of the drug while increasing the amount of drug which can accumulate in the tumor cells. However, the lack of an in-depth physico-chemical characterization of the liposomal TMZ formulations poses real problems, especially sample reproducibility, as well as the difficulty in developing a robust, scalable, and affordable preparation process. In addition, the extremely low number of clinical studies centered on liposomal TMZ points out the challenges of passing TMZ-liposomes from bench to bedside. Therefore, considering all advances and gaps of the results obtained so far and reviewed herein, it is obvious that including TMZ in a liposomal (nano)carrier is a feasible approach to provide realistic nanomedicines that are both pharmaceutically suitable and therapeutically valuable for GBM management, but more intense research efforts are needed in the years to come.

## Figures and Tables

**Table 1:** Commonly used (phospho)lipids for liposome preparation.

Head Group Substituent	(Phospho)lipid	Abbreviation	Molecular Weight (g·mol^−1^)	C/U	T_C_ (°C)	Charge (pH = 7)
**Choline**	Dilauroyl phosphatidylcholine	DLPC	621.83	12/0	−2	**Zwitteriionic/Neutral**
	Dimyristoyl phosphatidylcholine	DMPC	677.93	14/0	24	
	Dipalmitoyl phosphatidylcholine	DPPC	805.48	16/0	41	
	Distearoyl phosphatidylcholine	DSPC	790.15	18/0	55	
	Dioleoyl phosphatidylcholine	DOPC	786.11	18/1	−17	
**Ethanolamine**	Dilauroyl phosphatidylethanolamine	DLPE	579.75	12/0	29	
	Dimyristoyl phosphatidylethanolamine	DMPE	635.85	14/0	50	
	Dipalmitoyl phosphatidylethanolamine	DPPE	691.96	16/0	60	
	Distearoyl phosphatidylethanolamine	DSPE	748.07	18/0	74	
	Dioleoyl phosphatidylethanolamine	DOPE	744.03	18/1	−16	
**Glycerol**	Dilauroyl phosphatidylglycerol	DLPG	610.8	12/0	−3	**Anionic/Negative**
	Dimyristoyl phosphatidylglycerol	DMPG	666.9	14/0	23	
	Dipalmitoyl phosphatidylglycerol	DPPG	744.95	16/0	41	
	Distearoyl phosphatidylglycerol	DSPG	779.1	18/0	55	
	Dioleoyl phosphatidylglycerol	DOPG	775.0	18/1	−18	
**Serine**	Dilauroyl phosphatidylserine	DLPS	645.74	12/0		
	Dimyristoyl phosphatidylserine	DMPS	679.9	14/0	35	
	Dipalmitoyl phosphatidylserine	DPPS	736.0	16/0	51	
	Distearoyl phosphatidylserine	DSPS	792.1	18/0	68	
	Dioleoyl phosphatidylserine	DOPS	810.03	18/1	−11	
-	Dioleoyl trimethylammonium-propane	DOTAP	698.54	18/1	<5	**Cationic/Positive**

**Table 2:** Physico-chemical properties investigated in papers discussed in this review.

Size (nm)	Surface Charge (mV)	pH	Morphology /Structure	PL Composition (mg/mL)	EE (%)	Stability	Cell Line	In Vivo	Delivery Way	Reference
203.4	−1.60	-	-	-	99.20	-	-	-	IV	[80]
160.0	~0	-	Sphere (TEM)	-	87.00	-	-	-	CED	[51]
185.0	-	-	Sphere (SEM)	-	90.30	-	-	-	-	[28]
157.0	-	6.46	Sphere (TEM)	-	35.45	-	-	Rabbit mouse	IV	[9]
120.0	−0.20	-	Sphere/ unilamellar (cryoTEM)	28 (HPLC)	(HPLC)	-	CNS-1 rat glioma cell	rat	CED	[52]
137	−12.10	-	-	-	45.10 (HPLC)	-	U87, GL261	Mice		[81]
137.4	−49.90	-	-	-	-	-	-			[82]
135.6	−26.26	-	Sphere/ unilamellar (cryoTEM)	-	56.11 (UV-Vis)	Size, EE% 93 months	-	Rats	IV	[2]
41.4	30.10	-	-	-	45.23	-	U87-luc2 U251 U87R	Mice	IV	[78]
196.5	30.50		Sphere (TEM)	HPLC UV-Vis FT-IR	53.58–66.25 (HPLC)	-	U87/TR	Rats	Intraperi-toneal	[47]
133.0	−5.68		Sphere (TEM)			Size, CMC	GL261	Mice	IV	[83]

## Data Availability

Not applicable.
